# Experimentally derived biomimetic chromatographic descriptors for drug-induced phospholipidosis liability prediction

**DOI:** 10.5599/admet.3460

**Published:** 2026-07-10

**Authors:** Chrysanthos Stergiopoulos, Valko Klara

**Affiliations:** 1 Laboratory of Process Analysis and Design, School of Chemical Engineering, National Technical University of Athens, Iroon Polytechniou 9, 157 75 Zografou, Athens, Greece; 2 Bio-Mimetic Chromatography Ltd, Business & Technology Centre, Bessemer Drive, Stevenage, Herts, SG1 2DX, United Kingdom

**Keywords:** Phospholipidosis, biomimetic chromatography, membrane affinity, cationic amphiphilic drugs, lysosomal trapping

## Abstract

**Background and purpose:**

Drug-induced phospholipidosis (PLD) is a complex intracellular liability commonly associated with the lysosomal accumulation of cationic amphiphilic drugs and remains an important concern in drug development because of its implications for safety, intracellular disposition, and compound prioritization. Although cationic amphiphilicity, membrane interactions, and lysosomal trapping are established features of PLD, experimentally accessible descriptors that capture PLD-relevant membrane affinity remain valuable for early-stage risk assessment. This study aimed to evaluate whether biomimetic chromatographic descriptors can improve the prediction and interpretation of PLD liability by providing experimentally derived information on membrane affinity and protein-binding-related distributional behavior.

**Experimental approach:**

A curated dataset of 65 compounds with experimentally reported PLD responses was analyzed using multiple linear regression and ordinal classification approaches. PLD potency was expressed as pEC₅₀, while ordinal models classified compounds into non-inducers, weak/moderate inducers, and strong inducers. Membrane affinity was quantified using immobilized artificial membrane chromatography, expressed as CHI IAM, while protein-related interactions were represented by human serum albumin affinity, log *k*_HSA_, and α_1_-acid glycoprotein affinity, log k_AGP_. Biomimetic chromatographic descriptors were compared with conventional physicochemical parameters, including log P and log D, to assess their relative predictive and mechanistic value.

**Key results:**

CHI IAM-containing models showed strong predictive performance for PLD potency, with external predictivity reaching Q^2^_ext_ = 0.823. CHI IAM alone also performed strongly, achieving Q^2^_ext_ = 0.821, indicating that membrane affinity captures a major component of PLD-relevant behavior. In ordinal classification models, CHI IAM alone provided robust discrimination of PLD severity, with a test accuracy of 0.769, macro F1 score of 0.778, and macro-AUC of 0.923. Protein-binding descriptors, particularly log k_AGP_, showed moderate predictive value but offered limited, model-dependent improvement once membrane affinity was considered. Conventional lipophilicity descriptors did not outperform the biomimetic membrane-affinity descriptor.

**Conclusion:**

Biomimetic chromatographic descriptors provide experimentally derived, mechanistically interpretable surrogates for assessing early-stage PLD liability. CHI IAM offers a practical approximation of membrane-affinity behavior relevant to lysosomal phospholipid accumulation and PLD risk. Protein-binding descriptors may provide complementary information related to distributional phenotypes, but their added value appears less consistent than that of membrane affinity. This approach extends beyond conventional lipophilicity-based QSAR by incorporating experimentally measured distribution-relevant properties and may support compound prioritization during drug discovery.

## Introduction

Drug-induced phospholipidosis is a lysosomal lipid storage disorder characterized by excessive intracellular accumulation of phospholipids and the formation of concentric lamellar bodies detectable by transmission electron microscopy [1,2]. The phenomenon has been observed across several tissues, including liver, lung, kidney, and nervous tissue, and remains relevant in drug development because it can complicate preclinical safety interpretation and compound prioritization [1-4]. Although PLD is often considered an adaptive and potentially reversible cellular response rather than a direct toxicological endpoint, its association with altered lipid metabolism, intracellular drug accumulation, and secondary cellular dysfunction underscores the importance of early identification of phospholipidogenic compounds in drug discovery [3,4].

The most widely accepted mechanistic framework for PLD involves cationic amphiphilic drugs, which combine lipophilicity with one or more protonatable amine groups [5-7]. These compounds can passively diffuse across cellular membranes and subsequently accumulate in acidic lysosomal compartments via ion trapping, in which protonation reduces their ability to diffuse back into the cytosol [5,6]. Lysosomal accumulation is further promoted by interactions with phospholipid-rich membranes, leading to drug-phospholipid complex formation, impaired phospholipid degradation, and inhibition or functional disruption of lysosomal phospholipases [4-6]. Previous studies have shown that PLD liability is influenced by the interplay between lipophilicity, basicity, and distribution-related properties, including volume of distribution [7,8]. However, simple rules based on calculated logarithm of the octanol-water partition coefficient (log *P*), the negative logarithm of the acid dissociation constant (p*K*_a_) or cationic amphiphilicity are not sufficient to explain all cases, because structurally diverse and non-classical CAD compounds may also induce phospholipid accumulation [8].

Several experimental approaches have therefore been developed to detect or predict PLD liability. Cell-based assays remain among the most biologically relevant methods because they directly assess intracellular phospholipid accumulation. In an early and influential study, Casartelli *et al.* [9] demonstrated the utility of a cell-based approach for assessing phospholipidogenic potential in pharmaceutical research and drug development. Subsequent fluorescence-based assays using probes such as NBD-PE and high-content imaging platforms further improved the quantitative assessment of intracellular lipid accumulation and enabled better distinction between PLD induction and cytotoxicity-related effects [10,11]. These methods provide valuable biological information and can support compound safety profiling; however, they are more resource-intensive than simple physicochemical or chromatographic approaches and may not always be optimal for rapid early-stage screening.

Non-cell-based methods have also been proposed to evaluate drug-phospholipid interactions as surrogate indicators of PLD risk. Fluorescence-based lipid interaction assays, including methods using Prodan and other probes, can detect drug-induced perturbation of lipid systems and have shown useful correlations with PLD-related endpoints [12]. Similarly, biochemical and physicochemical assays, combined with multivariate analysis, have been used to estimate the phospholipidosis-inducing potential [13]. These approaches support the concept that PLD is not governed by a single molecular property, but by the combined influence of amphiphilicity, ionization, membrane affinity, and intracellular distribution.

Chromatographic approaches have also been explored for early PLD screening. Jiang and Reilly proposed chromatographic methods for assessing phospholipidosis-inducing potential, showing that retention behavior in selected chromatographic systems could provide useful physicochemical information for early screening [14]. More specifically, IAM-related chromatographic approaches have already been connected to PLD. Zhao *et al.* developed a mixed phospholipid-functionalized monolithic column and reported that CHI IAM 7.4 values were highly correlated with drug-induced PLD potency in a set of marketed drugs. More recently, Wang *et al.* developed an acidic phospholipid-containing immobilized artificial membrane column designed to better mimic the negatively charged lysosomal phospholipid environment and improve prediction of drug-induced PLD potency. These studies clearly establish that IAM-based membrane-affinity measurements are relevant to PLD assessment. Therefore, the present study lies not in the first application of IAM chromatography to PLD, but in the systematic comparative evaluation of standard biomimetic chromatographic descriptors against conventional physicochemical descriptors within a unified modelling framework.

In parallel with experimental approaches, computational models have been developed for PLD prediction, including classical QSAR, rule-based methods, and machine-learning models [8,17-19]. These methods can support high-throughput compound screening and have demonstrated useful predictive performance. However, many models rely mainly on calculated descriptors, structural fingerprints, or lipophilicity/basicity rules [8,17-20]. While such descriptors are valuable, they do not directly measure membrane affinity, phospholipid interaction, or protein-binding-related distributional behaviour. This limitation is important because PLD is fundamentally a distribution-driven intracellular phenomenon involving membrane partitioning, lysosomal accumulation, and drug-phospholipid interactions [4-7,20].

Biomimetic chromatography provides experimentally derived descriptors that can complement conventional calculated physicochemical parameters. Immobilized artificial membrane chromatography quantifies the affinity of compounds for phospholipid-like environments and has been widely used as a surrogate for membrane partitioning, permeability, tissue binding, and related ADMET properties [21-24]. Human serum albumin and α_1_-acid glycoprotein chromatography provide additional descriptors of plasma protein binding and systemic distribution [21-24]. These chromatographic measurements do not directly reproduce the acidic lysosomal environment; rather, they provide experimentally accessible surrogates for drug-membrane and drug-protein interactions that influence compound disposition. In the context of PLD, CHI IAM is particularly relevant because membrane affinity and phospholipid interaction are central features of lysosomal phospholipid accumulation. Protein-binding descriptors may provide complementary information about distributional phenotypes, especially for basic and amphiphilic compounds, although their mechanistic role in PLD should be interpreted with caution.

Building on previous PLD screening and IAM-based studies [14-16], the present work evaluates standard biomimetic chromatographic descriptors, including the chromatographic hydrophobicity index measured by immobilized artificial membrane chromatography (CHI IAM), the logarithmic retention factor on human serum albumin chromatography (log *k*_HSA_), and the logarithmic retention factor on α₁-acid glycoprotein chromatography (log *k*_AGP_), alongside conventional physicochemical descriptors in a curated dataset of compounds with experimentally reported PLD responses. The study compares these biomimetic descriptors with log *P*, the logarithm of the pH-dependent distribution coefficient (log *D*), molecular size, hydrogen-bonding descriptors, and ionization fractions to determine whether experimentally measured membrane affinity and protein-binding descriptors provide additional predictive and mechanistic value beyond conventional lipophilicity-based QSAR. In addition, both continuous and ordinal modelling strategies are applied. Multiple linear regression is used to model PLD potency, expressed as pEC₅₀, *i.e.* the negative logarithm of the EC₅₀ value expressed in molar units, where EC₅₀ denotes the concentration producing half-maximal intracellular phospholipid accumulation under the assay conditions. Ordinal regression is used to classify compounds into ordered PLD severity categories, namely non-inducers, weak/moderate inducers, and strong inducers.

Accordingly, the aim of this study was to reassess and extend the role of biomimetic chromatographic descriptors in PLD liability prediction using a comparative ADMET modelling framework. By integrating experimentally derived membrane-affinity and protein-binding descriptors with continuous potency modelling and ordinal severity classification, the study seeks to clarify whether CHI IAM and related biomimetic descriptors can serve as practical, interpretable surrogates for PLD-relevant distributional behaviour. This approach is intended not to replace cell-based PLD assays, but to provide an early-stage decision-support tool to identify compounds with elevated liability for phospholipidosis and to prioritize them for further experimental evaluation.

## Experimental

### Phospholipidosis data collection

The phospholipidosis dataset was compiled from previously published *in vitro* studies that employed fluorescence-based and high-content screening assays to detect drug-induced phospholipid accumulation [9-11,25-27]. The dataset included compounds evaluated in mammalian cell systems commonly used for PLD screening, including hepatocyte-derived HepG2 cells and macrophage models such as RAW264.7, I-13.35, and primary mouse macrophages [25-27]. These models are relevant because of their sensitivity to lysosomal phospholipid accumulation and their established use for identifying phospholipidosis hazards. PLD induction was assessed using fluorescent phospholipid probes, including NBD-PE and LipidTOX™, which accumulate in lysosomal compartments and serve as experimental surrogates for intracellular phospholipid accumulation [10,11,25,26]. Quantitative endpoints were derived from fluorescence intensity measurements obtained by automated imaging or plate-based detection systems and reflected the extent of intracellular phospholipid accumulation. For each compound, concentration-response data were used to derive potency values expressed as EC₅₀. When multiple experimental values were available for the same compound, a predefined selection hierarchy was applied. First, priority was given to quantitative EC₅₀ values derived from concentration-response experiments that directly measured intracellular phospholipid accumulation using fluorescence-based or high-content PLD assays. Second, values were preferred when the concentration-response relationship was complete, and the reported potency value was clearly derived from a fitted dose-response curve rather than from a single-concentration response or qualitative classification. Third, among otherwise comparable values, preference was given to measurements obtained under assay conditions most consistent with the rest of the curated dataset, including comparable mammalian cell-based PLD endpoints and clearly reported exposure-response information. Values were not retained when the endpoint was not directly related to intracellular phospholipid accumulation, when the concentration-response information was incomplete, or when interpretation was clearly confounded by cytotoxicity or insufficient methodological detail. To enable direct comparison with physicochemical and chromatographic descriptors, EC₅₀ values reported in micromolar units were converted to molar units and transformed to a negative logarithmic scale: pEC₅₀ = -log₁₀ (EC₅₀ / M). Higher pEC₅₀ values, therefore, indicate greater PLD-inducing potency. The complete list of compounds, together with their corresponding potency values and PLD classifications, is provided in Supplementary material, Table S1.

### Phospholipid binding (immobilized artificial membrane chromatography)

Immobilized artificial membrane chromatography was used to characterize the membrane affinity of the studied compounds. IAM stationary phases contain phosphatidylcholine analogs covalently immobilized on silica and provide a biomimetic environment that captures key aspects of drug-membrane interactions, including hydrophobic, polar and electrostatic contributions [21,23,24]. Chromatographic analyses were performed using an IAM.PC.DD2 column under gradient elution conditions, with ammonium acetate buffer at pH 7.4 as the aqueous phase and acetonitrile as the organic modifier. Retention times were converted to chromatographic hydrophobicity index (CHI) values, expressed as CHI IAM, using calibration with reference compounds in accordance with established biomimetic chromatography methodology [21,23,24]. The calibration showed excellent linearity, with *R*^2^ > 0.99. The resulting CHI IAM values were used as experimentally derived descriptors of membrane affinity.

### Human serum albumin and α_1_-acid glycoprotein chromatography

Protein-binding-related distributional properties were characterized using biomimetic chromatography on immobilized human serum albumin (HSA) and α_1_-acid glycoprotein (AGP) stationary phases. These chromatographic descriptors were included to evaluate whether protein-binding-related retention provides complementary information to membrane-affinity measurements in PLD prediction. Chromatographic separations were performed using gradient elution with ammonium acetate buffer at pH 7.4 and isopropanol as the organic modifier. Retention data were converted to logarithmic retention factors (log *k*_HSA_ and log *k*_AGP_) using calibration curves derived from reference compounds, following established biomimetic chromatography procedures [22-24]. The calibration curves showed excellent linearity, with *R*^2^ > 0.99. The resulting descriptors provide experimentally derived measures of drug-protein interactions related to systemic distribution and plasma protein-binding behaviour.

The experimentally determined biomimetic chromatographic descriptors, including CHI IAM, log *k*_HSA_, and log *k*_AGP_, are provided in Supplementary material, Table S2.

### Physicochemical descriptors

A set of conventional physicochemical descriptors was collected for all compounds to enable comparison with biomimetic chromatographic parameters and to support modelling analyses. These descriptors included lipophilicity parameters (log *P* and log *D* at pH 7.4, log *D*_7.4_), MW, TPSA, and charge-related descriptors, namely the fractions of positively charged species, negatively charged species, and zwitterionic forms at physiological pH. Hydrogen-bonding properties were described using both structural and solvation-based descriptors. Specifically, HBD and HBA counts were included as simple structural descriptors, while Abraham’s solvation parameters for hydrogen bond acidity (*A*) and basicity (*B*) were also considered, providing a more quantitative representation of intermolecular hydrogen-bonding interactions. The combined use of these descriptors enables capture of both the presence and strength of hydrogen-bonding capacity, which is known to influence membrane partitioning, protein binding, and intracellular distribution. All physicochemical descriptors were calculated using the Percepta platform (ACD/Labs, Advanced Chemistry Development, Toronto, Canada) [28], which provides standardized computational estimates of molecular properties relevant to drug disposition. A complete list of calculated descriptors for all compounds is provided in Supplementary Material Table S3. These descriptors were included to evaluate the performance of traditional property-based models against biomimetic chromatographic descriptors and to assess their contribution to predicting phospholipidosis potential.

### Dataset splitting and characterization

The dataset, comprising 65 compounds, was divided into a training set (*n* = 52) and an independent external test set (*n* = 13) using response-stratified sampling to ensure approximately 80:20 coverage of the pEC₅₀ range. This approach was selected to preserve the distribution of biological activity across both sets, thereby avoiding bias toward specific activity regions and supporting the development of predictive and generalizable models [29,30]. Descriptive statistics (mean, median, standard deviation, minimum, maximum, and range) were calculated to characterize the distribution of pEC₅₀ values, while compounds were split ordinally and their activity class distribution was assessed to ensure balanced representation between the training and test sets (Table S1 and Table S4). The distribution of pEC₅₀ values was examined using descriptive statistics and frequency histograms (Figure S1) to assess the consistency of the activity range and variability between the two sets. In parallel, the distribution of ionization states was assessed by classifying compounds into charge categories (base, weak base, neutral, acid, zwitterion) (Figure S2), as charge-related properties are known to influence membrane interactions and transporter behaviour. To evaluate the representativeness of the training and test sets in chemical space, PCA was performed on all biomimetic and physicochemical descriptors. This analysis was employed to ensure that the external test set falls within the descriptor space defined by the training set, thereby supporting reliable external validation and minimizing extrapolation.

### Multiple linear regression

MLR models were developed to investigate the relationships among biomimetic chromatographic descriptors, physicochemical properties and phospholipidosis potential, expressed as pEC₅₀ [29,30]. These models used the training and external test sets described above and were fitted by ordinary least squares regression. Descriptor selection was based on mechanistic relevance, coefficient-level statistical significance, and avoidance of multicollinearity. Individual regression coefficients were evaluated using t-tests, and descriptors were retained in the presented MLR equations only when statistically significant at *p* < 0.05. Multicollinearity among descriptors was assessed using variance inflation factors (VIFs). For each descriptor, VIF was calculated by Equation (1):



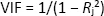

(1)


where *R*_j_^2^ is obtained by regressing that descriptor against the remaining descriptors in the model.

Thus, VIF quantifies how much the variance of a regression coefficient is inflated by correlation with other predictors. Models with VIF values below 5 were considered acceptable and were retained. Model performance was evaluated using the coefficient of determination (*R*^2^), adjusted *R*^2^ (*R*^2^_adj_), and the *F*-statistic. Internal validation was performed using 5-fold cross-validation, yielding the cross-validated coefficient (*Q*^2^_cv_) and the RMSE_cv_. External predictive performance was assessed on the test set by calculating *Q*^2^_ext_ and RMSEP. Definitions and formulas for the validation metrics used in this study, including *Q*^2^_cv_, *Q*^2^_ext_, RMSE_cv_ and RMSEP, follow established QSAR validation recommendations [29,30].

### Partial least squares

PLS regression was applied as a complementary multivariate method to assess the potential impact of descriptor interdependence on model performance. PLS is inherently robust to collinearity, as it projects the original variables onto latent components that maximize covariance with the response variable [31]. Models with suspected descriptor interdependence were developed using the same training and external test sets as those used in the MLR analysis. The optimal number of latent components was determined based on cross-validation (5-fold), selecting the model that maximized predictive performance (*Q*^2^_cv_) while avoiding overfitting. Model performance was evaluated using R^2^ for the training set, *Q*^2^_cv_ for internal validation, and *Q*^2^_ext_ for external validation, along with the corresponding RMSE values. The PLS analysis was used solely to confirm the robustness of the MLR findings with respect to descriptor interdependence.

### Applicability domain

The applicability domain of the developed MLR models was evaluated using the leverage approach (Williams plot) [30]. Leverage values (*h*_i_) were calculated from the hat matrix, and a warning leverage (h*) was defined as h* = 3(*p* + 1)/*n*, where p is the number of model parameters, and n is the number of training compounds. Standardized residuals were calculated based on the standard deviation of the training residuals. Compounds with *h*_i_ > h* were considered structurally influential, while those with standardized residuals outside ±3 were identified as response outliers.

### Ordinal regression modelling

To account for the ordered nature of phospholipidosis severity, ordinal regression models were developed using a proportional-odds logistic regression framework [32]. This approach is appropriate when the response variable consists of ordered categories rather than independent nominal classes. In the present study, compounds were categorized according to pEC₅₀ into non-inducers (pEC₅₀ < 4), weak/moderate inducers (4 ≤ pEC₅₀ ≤ 5), and strong inducers (pEC₅₀ > 5). These thresholds correspond to PLD-induction EC₅₀ values of >100 μM, 10 to 100 μM and <10 μM, respectively, where EC₅₀ denotes the concentration producing half-maximal intracellular phospholipid accumulation under the assay conditions. The thresholds were selected as pragmatic, order-of-magnitude potency categories for early PLD risk stratification rather than as regulatory cutoffs. This categorization separates compounds that show no or only low-potency PLD induction at high micromolar concentrations from those that produce phospholipid accumulation at lower micromolar concentrations. The use of concentration-response-derived PLD potency values is consistent with previous fluorescence-based and high-content *in vitro* phospholipidosis screening studies [9-11,25-27]. The model estimates the cumulative probability that a compound belongs to a given class or a lower class, assuming that the effect of each descriptor is constant across class thresholds.

Model fit was assessed using log-likelihood, AIC, BIC and McFadden’s pseudo-*R*^2^ [32-35]. Log-likelihood is the logarithm of the probability of the observed classification data under the fitted model, given the estimated model parameters [32,35]. Higher, or less negative, LL values indicate better model fit, although LL was interpreted alongside AIC and BIC, as it is affected by model complexity. AIC and BIC were calculated by Equations (2) and (3) [33,34]:



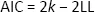

(2)




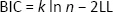

(3)


where LL is the maximized log-likelihood, *k* is the number of estimated model parameters, and *n* is the number of observations.

McFadden’s pseudo-*R*^2^ was calculated by Equation (4) [35]:



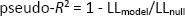

(4)


where LL_model_ and LL_null_ are the log-likelihoods of the fitted and intercept-only models, respectively.

McFadden’s pseudo-*R*^2^ expresses the improvement of the fitted model relative to a null model containing only intercept terms; it is not directly equivalent to the *R*^2^ used in linear regression but provides an indication of relative model fit. Predictive performance was evaluated by assigning each compound to the class with the highest predicted probability. Classification accuracy was calculated as the proportion of correctly classified compounds. Because the three PLD classes were not treated as interchangeable and class balance is important for risk stratification, macro-averaged F1 score (measure of the harmonic mean of precision and recall) was also calculated as the unweighted mean of the class-specific F1 scores. Thus, macro-F1 gives equal weight to non-inducers, weak/moderate inducers, and strong inducers.

Receiver operating characteristic analysis was performed using a one-*vs*-rest strategy for multiclass classification. In this approach, each PLD class is considered in turn as the positive class, with the remaining two classes combined as the negative class. The corresponding AUC values therefore describe the model's ability to discriminate each class from all others. Macro-AUC was calculated as the unweighted average of the class-specific one-*vs*-rest AUC values. Confusion matrices were generated for both the training and external test sets to evaluate the distribution of correct and misclassified classifications across ordered PLD categories.

### Model comparison strategy

The performance of biomimetic, protein-binding, and conventional physicochemical models was systematically compared across both regression and classification frameworks. Emphasis was placed on evaluating whether biomimetic chromatographic descriptors (CHI IAM, log *k*_HSA_, log *k*_AGP_) offer improved predictive power over traditional descriptors such as log *P* and log *D*, while considering both statistical robustness and mechanistic interpretability. Model comparison was based on a combination of statistical robustness (*Q*^2^_cv_, *Q*^2^_ext_), predictive accuracy (RMSEP, classification metrics), and mechanistic interpretability.

For regression models, predefined acceptance criteria were used to support model interpretation. Retained descriptors were required to show coefficient-level statistical significance (*p* < 0.05), and multicollinearity was considered acceptable when VIF values were below 5. Models interpreted as predictive were expected to show stable internal validation and external predictive performance, as indicated by positive *Q*^2^_cv_ and *Q*^2^_ext_ values, preferably above 0.5, together with low RMSE_cv_ and RMSEP relative to the dataset’s pEC₅₀ range. Applicability-domain assessment was also required, with most compounds expected to fall below the warning leverage threshold and within ±3 standardized residuals. Models that did not satisfy these predictive criteria were retained only as comparative or baseline models, not as primary predictive models. For ordinal regression models, acceptable performance required improvement over the null model, assessed by McFadden’s pseudo-*R*^2^, together with balanced class performance based on macro-F1 and discrimination ability based on macro-averaged one-*vs*-rest AUC. Ordinal models were considered useful for risk stratification when they exhibited balanced classification performance and avoided systematic confusion between distant PLD classes.

All statistical analyses and data visualization were performed using Python with the statsmodels and scikit-learn libraries.

## Results and discussion

### Dataset partitioning and distribution

The training and external test sets (52 and 13 compounds, respectively) showed comparable descriptive statistics, indicating a balanced partitioning of the dataset (Table S4). The training and test sets exhibited similar central tendency and dispersion (mean pEC₅₀: 4.63 *vs.* 4.51; median: 4.85 *vs.* 4.73; SD: 0.706 *vs.* 0.765), as well as overlapping activity ranges (3.10 to 5.87 for training and 3.00-5.41 for test). The distribution of pEC₅₀ values (Figure S1) further supports this, showing consistent coverage across the activity spectrum without apparent major gaps or clustering. Importantly, the class distribution of phospholipidosis (PLD) induction was maintained between the two subsets (Table S4), with similar proportions of non-inducers (25.0 *vs.* 30.8 %), weak/moderate inducers (38.5 *vs.* 38.5 %), and strong inducers (36.5 *vs.* 30.8 %). Likewise, the distribution of ionization states remained consistent across the training and test sets (Figure S2), ensuring that key physicochemical characteristics relevant to lysosomal accumulation were not biased by the split.

**Figure 1.**
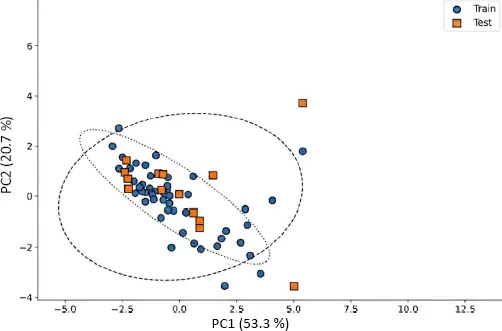
PCA plot illustrating the chemical-space coverage of the training and external test sets. Blue circles represent training compounds and orange squares represent external test compounds. The broader dashed confidence ellipse corresponds to the training set, whereas the smaller dashed confidence ellipse corresponds to the external test set

The representativeness of the split was further evaluated through principal component analysis (Figure 1). The first two principal components accounted for a substantial proportion of the total variance (PC1: 53.3 %, PC2: 20.7 %), indicating that the projection captures the major sources of variability in the dataset. The PCA plot shows that the test set compounds are well distributed within the chemical space defined by the training set, with no systematic separation or extrapolation beyond the training set's boundaries.

The strong overlap between the two subsets, as illustrated by the clustering and confidence ellipses, supports that the external test compounds are located within the descriptor space sampled by the training set, reducing the likelihood of extrapolative predictions.

### Multiple linear regression models for phospholipidosis prediction

A series of MLR models was developed to evaluate the ability of conventional physicochemical and biomimetic chromatographic descriptors to predict PLD potency, expressed as pEC₅₀. The performance of representative models is summarized in Table 1, while the full set of developed models and statistical parameters is provided in Supplementary Table S5.

**Table 1.** Performance of representative MLR models for prediction of phospholipidosis potency, expressed as pEC₅₀

Model	Equation	*R* ^2^	*Q* ^2^_cv_	*Q* ^2^_ext_	RMSEP
CHI IAM	pEC₅₀ = 3.077 + 0.038·CHI IAM	0.699	0.657	0.821	0.315
CHI IAM + *f*_neg_	pEC₅₀ = 3.22 + 0.035·CHI IAM − 0.346·*f*_neg_	0.724	0.689	0.823	0.313
CHI IAM + log *k*_AGP_	pEC₅₀ = 3.58 + 0.022·CHI IAM + 0.317·log *k*_AGP_	0.740	0.700	0.766	0.360
log *D*_7.4_	pEC₅₀ = 4.33 + 0.182·log *D*_7.4_	0.365	0.301	0.676	0.423
log *D*_7.4_ + *f*_pos_	pEC₅₀ = 3.68 + 0.177·log *D*_7.4_ + 0.878·*f*_pos_	0.579	0.479	0.761	0.364
log *k*_AGP_	pEC₅₀ = 4.36 + 0.655·log *k*_AGP_	0.677	0.648	0.539	0.506
log *k*_AGP_ + *f*_pos_	pEC₅₀ = 4.06 + 0.594·log *k*_AGP_ + 0.444·*f*_pos_	0.726	0.665	0.586	0.479

All descriptor coefficients in the MLR equations shown in Table 1 were statistically significant according to coefficient-level *t*-tests (*p* < 0.05). Descriptors that did not meet this criterion were not retained in the presented models.

According to these predefined validation criteria, the CHI IAM-based models satisfied the main requirements for predictive interpretation. The CHI IAM model showed strong internal and external performance, with *Q*^2^_cv_ = 0.657 and *Q*^2^_ext_ = 0.821, while the CHI IAM + *f*_neg_ model showed slightly improved performance, with *Q*^2^_cv_ = 0.689 and *Q*^2^_ext_ = 0.823. Both models also showed acceptable RMSEP values relative to the dataset's pEC₅₀ range. The CHI IAM + log *k*_AGP_ model showed the highest goodness of fit, but its external predictive performance was lower than that of CHI IAM alone and CHI IAM + *f*_neg_. By contrast, some conventional descriptor models, such as log *D*_7.4_ alone, did not meet the same internal validation criterion and were therefore interpreted primarily as baseline comparators rather than preferred predictive models.

Initial models based on conventional lipophilicity descriptors, including log *P* and log *D*, showed moderate predictive performance, indicating that bulk lipophilicity alone is insufficient to capture PLD potency in the studied dataset. The inclusion of the positively charged fraction (*f*_pos_) improved these conventional models, which is consistent with the established role of cationic amphiphilic drug-like behavior in PLD induction [4-8]. Cationic amphiphilic drugs typically combine a lipophilic domain with protonatable basic functionality, enabling membrane permeation, subsequent protonation, and lysosomal sequestration in acidic intracellular compartments [5,6]. Thus, the improvement observed after inclusion of *f*_pos_ is consistent with the contribution of ionization state to lysosomal accumulation and PLD liability.

By comparison, biomimetic chromatographic descriptors showed stronger predictive performance than conventional lipophilicity descriptors in this dataset. The CHI IAM descriptor, which reflects interaction with phospholipid-like surfaces, showed a strong association with PLD potency (*R*^2^ = 0.699, *Q*^2^_ext_ = 0.821) and outperformed the single conventional descriptors evaluated in Table 1. This observation is mechanistically plausible because PLD involves accumulation and perturbation within phospholipid-rich intracellular compartments, whereas octanol/water partitioning provides only a simplified representation of molecular lipophilicity [4-6,20]. IAM stationary phases provide an experimentally accessible phospholipid-like environment, and IAM retention has been used as a surrogate for membrane affinity, membrane partitioning, and related ADMET properties [21,23,24]. Previous IAM-based PLD studies further support the relevance of chromatographic membrane-affinity measurements for assessing phospholipidosis [14-16]. In this context, the strong performance of CHI IAM suggests that experimentally measured membrane affinity captures PLD-relevant information not fully represented by calculated log *P* or log *D*.

The addition of charge-related information further refined the CHI IAM-based models. The CHI IAM + *f*_neg_ model showed the highest external predictive performance among the representative MLR models (*Q*^2^_ext_ = 0.823; RMSEP = 0.313), although the improvement over CHI IAM alone was small. This suggests that ionization-related descriptors may provide complementary information, but that CHI IAM already captures a substantial part of the PLD-relevant signal. Although PLD is classically associated with cationic amphiphilic compounds, the broader ionization profile of a molecule may still influence membrane association, intracellular distribution, and apparent PLD potency.

The CHI IAM + log *k*_AGP_ model provided the highest goodness-of-fit among the representative models (*R*^2^ = 0.740), but its external predictivity was lower than that of CHI IAM alone and CHI IAM + *f*_neg_ (*Q*^2^_ext_ = 0.766 *vs.* 0.821-0.823). This pattern indicates that log *k*_AGP_ may capture additional distribution-related information within the training set, but its added predictive value was not consistently reflected in the external test set. From a mechanistic perspective, AGP binding should not be interpreted as a direct driver of PLD. Rather, log *k*_AGP_ can be viewed as an experimentally derived descriptor that reflects physicochemical and distributional features common to many basic and amphiphilic drugs [22,36]. Therefore, its contribution is best interpreted as a complementary distribution-related signal rather than as an independent mechanistic cause of phospholipid accumulation.

Because IAM retention and AGP binding are both influenced by amphiphilicity, ionization, and molecular size, these descriptors are not expected to be fully independent. This behaviour is consistent with the overlapping physicochemical determinants of membrane affinity and plasma protein binding in ADMET-related descriptor sets [21-24,36]. VIF analysis (Supplementary material Table S5) indicated acceptable VIF values, suggesting that multicollinearity was not problematic in the retained models. To further assess the effect of descriptor interdependence, partial least squares regression was applied as a complementary analysis (Supplementary material Table S6). The PLS results indicated that a single latent component captured most of the predictive variance, whereas additional components did not substantially improve model performance. These findings support the interpretation that the predictive information is partly shared across related distributional descriptors, and that CHI IAM provides a practical experimental surrogate for this membrane-affinity-dominated descriptor space.

The comparatively strong performance of log *k*_AGP_ alone (*R*^2^ = 0.677) and the weaker performance of log k_HSA_ alone (*R*^2^ = 0.313) are consistent with the tendency of AGP to bind many basic drugs, whereas HSA more commonly contributes to the binding of neutral and acidic compounds [22,36]. Although incorporating *f*_pos_ and *f*_neg_ improved the performance of log *k*_HSA_-based models, these models remained less predictive than the strongest CHI IAM-based models. This supports the conclusion that membrane-affinity information is central to the present modelling framework, whereas protein-binding descriptors provide more limited, model-dependent complementary information.

**Figure 2.**
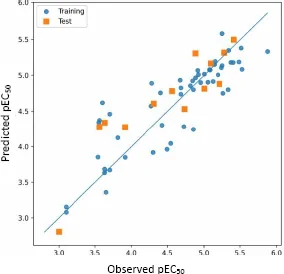
Observed *versus* predicted pEC₅₀ values for the MLR model based on CHI IAM and log *k*_AGP_. Training and external test sets are shown. The solid line represents the ideal y = x relationship. Model performance: *R*^2^ = 0.740, *Q*^2^_cv_ = 0.700, *Q*^2^_ext_ = 0.766 and RMSEP = 0.360

This interpretation is illustrated in Figure 2, which shows the agreement between observed and predicted pEC₅₀ values for the CHI IAM + log *k*_AGP_ model in the training and external test sets.

**Figure 3.**
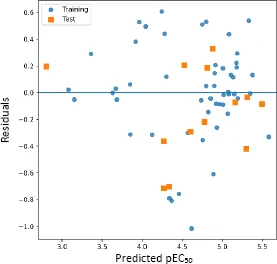
Residuals plot for the CHI IAM + log *k*_AGP_ MLR model. Residuals, calculated as observed − predicted pEC₅₀, are plotted against predicted pEC₅₀ values for both training and test compounds

The diagnostic plots further support the stability of the selected biomimetic model. In Figure 3, the residuals of the CHI IAM + log *k*_AGP_ model are distributed around zero without obvious systematic curvature, suggesting no major visual evidence of bias.

**Figure 4.**
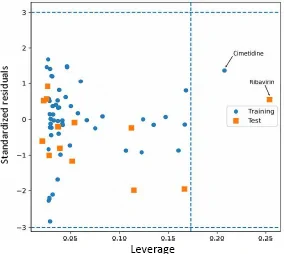
Williams plot for the CHI IAM + log *k*_AGP_ MLR model. Standardized residuals are plotted against leverage values for training and external test compounds. The horizontal dashed lines indicate the ±3 standardized residual limits, while the vertical dashed line represents the leverage warning threshold. Cimetidine and Ribavirin exceeded the leverage threshold and are labelled in the plot

Figure 4 shows that most compounds fall within the model applicability domain, with leverage values below the warning threshold and standardized residuals within ±3. Together with the VIF analysis and statistically significant regression coefficients reported in Supplementary material Table S5, these diagnostics support the use of the model for interpretation within the chemical space covered by the dataset.

The Williams plot identified two compounds outside the leverage warning threshold: Cimetidine and Ribavirin. Importantly, neither compound exceeded the ±3 standardized residual limits, indicating that they should be regarded as high-leverage compounds rather than response outliers. Ribavirin is a highly polar nucleoside analog with very low biomimetic membrane and AGP retention, placing it at the low-affinity edge of the descriptor space and outside the main region occupied by typical cationic amphiphilic PLD inducers. Cimetidine is also a comparatively polar and structurally atypical compound, combining imidazole, cyanoguanidine, and thioether functionalities, with relatively low membrane affinity but measurable PLD activity. These structural features may explain why both compounds occupy influential positions in the model space. However, their residuals remained within acceptable limits, suggesting that they define the edge of the model's applicability domain rather than undermining it.

The comparison between descriptor classes indicates that PLD potency is not adequately described by conventional lipophilicity metrics alone. The stronger performance of CHI IAM-based models is consistent with the involvement of membrane-affinity and distribution-related processes in PLD induction. Positive charge and AGP-related retention may modulate this behaviour, but their contributions appear secondary or context-dependent compared with the membrane-affinity signal captured by CHI IAM. This interpretation is consistent with the accepted mechanistic framework of PLD, in which lysosomal sequestration, drug-phospholipid interactions, and altered phospholipid turnover arise from the interplay of amphiphilicity, ionization, and membrane partitioning [4-7].

Mechanistically, these findings are consistent with lysosomal trapping of cationic amphiphilic compounds. After passive diffusion across cellular membranes, protonatable molecules can become increasingly ionized in the acidic lysosomal environment, reducing their ability to diffuse back across the membrane and promoting intracellular accumulation [5,6]. The resulting enrichment of amphiphilic basic compounds in lysosomes can favour interactions with phospholipid-rich structures, thereby contributing to the disruption of lysosomal lipid homeostasis [4-6]. Within this framework, CHI IAM should be interpreted as an experimentally derived surrogate of membrane affinity rather than as a direct measurement of lysosomal accumulation. Its predictive performance supports its use as a practical descriptor for early PLD liability assessment, particularly when combined with appropriate validation and applicability-domain analysis.

### Ordinal regression analysis of phospholipidosis classification

Following the development of continuous models for PLD potency using MLR, an ordinal regression approach was applied to evaluate whether the same descriptors could classify compounds into ordered PLD potency categories. This analysis complements the continuous regression framework by focusing on categorical risk stratification, which is particularly relevant for early-stage screening. In this context, reducing false-negative classifications is important, as a practical objective in early discovery is to flag compounds with elevated PLD liability for further evaluation or prioritization. The ordinal regression analysis was not intended to replace the continuous MLR equations or to provide a more readily transferable predictive equation; rather, it was used as a complementary analysis to examine whether the same descriptors could support categorical PLD risk stratification.

As summarized in Table 2, the CHI IAM model achieved the most balanced ordinal classification performance among the representative models, with a test accuracy of 0.769, a macro-F1 score of 0.778, and a macro-AUC of 0.923. This result is consistent with the full ordinal model comparison reported in Supplementary Table S7 and with the continuous MLR analysis, indicating that the same descriptor hierarchy was preserved when PLD liability was expressed as ordered potency classes rather than as continuous pEC₅₀ values. CHI IAM remained statistically significant in the relevant ordinal models, whereas additional descripttors such as *f*_neg_ or log *k*_AGP_ did not consistently improve classification performance or provide significant additional contributions. Protein-binding and conventional lipophilicity descriptors showed useful but less consistent classification behaviour, particularly when evaluated by macro-F1 and class assignment accuracy. Therefore, the ordinal models are interpreted as supportive risk-stratification tools rather than as an independent source of mechanistic evidence.

**Table 2.** Performance of representative ordinal regression models for phospholipidosis classification.

Model class	Model	Test accuracy	Test macro F1	Test AUC (macro OVR)	McFadden’s *R*^2^
Membrane-binding	CHI IAM	0.769	0.778	0.923	0.440
Protein-binding	log k_AGP_	0.769	0.761	0.861	0.377
Conventional	log D_7.4_	0.692	0.689	0.915	0.169

Using the predefined ordinal-model criteria, the CHI IAM model showed the most balanced validation profile, with McFadden’s pseudo-*R*^2^ = 0.440, macro-F1 = 0.778, and macro-AUC = 0.923. These values indicate meaningful improvement over the null model, balanced class-level performance, and strong one-*vs*-rest discrimination. The log *k*_AGP_ and log *D*_7.4_ models showed useful but less consistent profiles, particularly when macro-F1 and McFadden’s pseudo-*R*^2^ were considered together.

**Figure 5.**
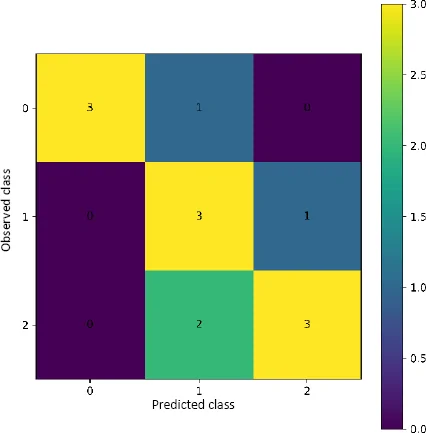
Confusion matrix for the ordinal regression model based on CHI IAM, evaluated on the external test set

The confusion matrix for the CHI IAM model (Figure 5) provides additional insight into classification behaviour. Misclassifications occurred mainly between adjacent PLD classes rather than between distant categories, consistent with the ordered nature of the endpoint. The corresponding training-set confusion matrix is provided in Figure S3 and shows a similar pattern, with no direct confusion between non-inducers and strong inducers.

**Figure 6.**
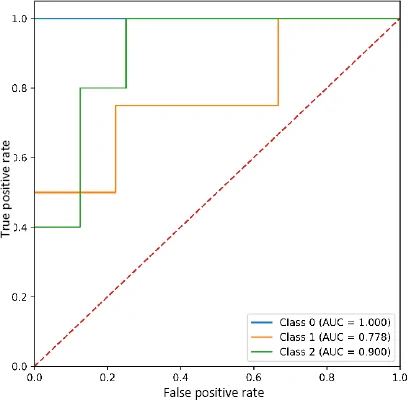
Multiclass receiver operating characteristic curves for the CHI IAM ordinal regression model evaluated on the external test set. ROC curves were generated using a one-*vs*-rest approach, in which each PLD class was considered separately as the positive class and the remaining two classes were combined as the negative group. The class-specific AUC values therefore describe the model's ability to discriminate non-inducers, weak/moderate inducers, and strong inducers from all other compounds

The one-*vs*-rest ROC analysis (Figure 6) showed stronger discrimination for the non-inducer and strong-inducer classes than for the weak/moderate inducer class. The corresponding training-set ROC curves are provided in Figure S4. These results support the use of ordinal regression as a complementary classification analysis, while the main mechanistic interpretation remains based on the continuous MLR models.

**Figure 7.**
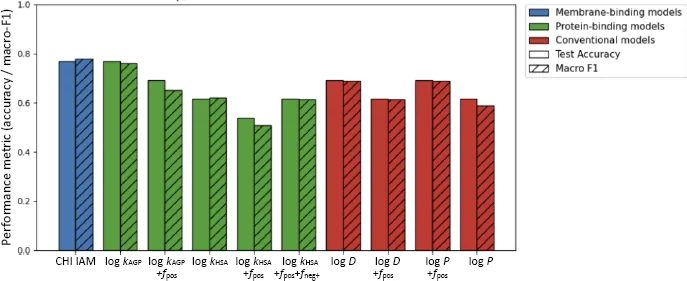
Comparative performance of ordinal regression models based on membrane-binding, protein-binding, and conventional descriptors (significant coefficients only). Test accuracy and macro F1 score are shown for each model

A broader comparison across descriptor families (Figure 7) further illustrates that the ordinal classification results follow the same trend observed in the continuous MLR models, with CHI IAM showing the most balanced performance among the representative descriptor classes.

The ordinal regression analysis confirmed the same descriptor hierarchy observed in the continuous MLR models. CHI IAM provided the most consistent performance, whereas protein-binding and conventional lipophilicity descriptors showed more model-dependent behaviour. The model coefficient estimates and class thresholds reported in Supplementary Tables S8 and S9 further support the interpretability of the ordinal modelling framework within the chemical space represented by the dataset. Therefore, the ordinal analysis was retained as a complementary risk-classification exercise, while the main mechanistic interpretation was consolidated with the MLR-based discussion above. These models should be viewed as early-stage prioritization tools rather than replacements for cell-based PLD assays.

## Conclusions

In this study, biomimetic chromatographic descriptors were evaluated primarily through continuous regression modelling of PLD potency, with ordinal regression retained as a complementary risk-stratification analysis. Across these analyses, membrane affinity, represented by CHI IAM, emerged as the most informative and consistent descriptor within the studied dataset. CHI IAM-based models showed strong predictive performance in the continuous pEC₅₀ analysis and provided balanced classification performance in the ordinal regression analysis, supporting the relevance of experimentally measured membrane affinity for PLD liability assessment. The inclusion of additional descriptors, such as log *k*_AGP_ or ionization-related variables, yielded only limited, model-dependent improvements. This suggests that protein-binding and charge-related descriptors may provide complementary distributional information, but their added value appears less consistent once membrane affinity is explicitly accounted for. Conventional lipophilicity descriptors, including log *P* and log *D*, showed weaker and more variable performance, highlighting the limitation of relying solely on bulk lipophilicity to describe membrane-associated processes involved in PLD. Importantly, CHI IAM provides an experimentally accessible surrogate of membrane-affinity behaviour, which is relevant to drug-phospholipid interactions and intracellular distribution processes associated with PLD. Thus, biomimetic chromatography can complement conventional physicochemical descriptors by adding experimentally measured interaction information that is not fully captured by calculated lipophilicity parameters. Overall, the findings support the use of CHI IAM and related biomimetic chromatographic descriptors as practical, interpretable tools for early-stage PLD liability assessment and compound prioritization. The proposed approach may serve as a decision-support layer to identify compounds at elevated risk of phospholipidosis and to guide further experimental evaluation. Future studies using larger, more diverse datasets, harmonized experimental endpoints, and additional external validation will be important for further defining the applicability domain and translational value of this modelling framework.

## Supplementary material

Additional data are available at https://pub.iapchem.org/ojs/index.php/admet/article/view/3460, or from the corresponding author on request.



## Abbreviations

*A*: Abraham hydrogen bond acidity
ACD/Labs: advanced chemistry development
ADMET: absorption, distribution, metabolism, excretion and toxicity
AGP: α₁-acid glycoprotein
AIC: Akaike information criterion
AUC: area under the receiver operating characteristic curve
*B*: Abraham hydrogen bond basicity
BIC: Bayesian information criterion
CAD: cationic amphiphilic drug
CAS: Chemical Abstracts Service registry number
CHI IAM: chromatographic hydrophobicity index measured by immobilized artificial membrane chromatography
CV: cross-validation
EC₅₀: half-maximal effective concentration
*f*_neg_: fraction of negatively charged species
*f*_pos_: fraction of positively charged species
*f*_zw_: fraction of zwitterionic species
HBA: hydrogen bond acceptor
HBD: hydrogen bond donor
HSA: human serum albumin
IAM: immobilized artificial membrane
LL: log-likelihood
log *k*_AGP_: logarithmic retention factor on the α₁-acid glycoprotein stationary phase
log *k*_HSA_: logarithmic retention factor on the human serum albumin stationary phase
MLR: multiple linear regression
MW: molecular weight
OLS: ordinary least squares
OVR: one-*vs*-rest
PCA: principal component analysis
pEC₅₀: negative logarithm of EC₅₀ expressed in molar units
PLD: phospholipidosis
PLS: partial least squares
*Q* ^2^_cv_: cross-validated coefficient of determination
*Q* ^2^_ext_: external predictive coefficient of determination
QSAR: quantitative structure-activity relationship
*R* ^2^_adj_: adjusted coefficient of determination
RMSE: root-mean-square error
RMSE_cv_: root-mean-square error of cross-validation
RMSEP: root-mean-square error of prediction
ROC: receiver operating characteristic
TPSA: topological polar surface area
VIF: variance inflation factor
