# Supplementary material for: Experimentally derived biomimetic chromatographic descriptors for drug-induced phospholipidosis liability prediction
Source: ADMET DMPK. 2026 Jul 10;14:3460. doi: 10.5599/admet.3460 (PMC13499669; doi:10.5599/admet.3460)
Supplement: Supplementary file 1 [file ADMET-14-3460-S1.pdf]

ADMET & DMPK **14** (2026) S3460

Open Access : ISSN : 1848-7718
http://www.pub.iapchem.org/ojs/index.php/admet

Supplementary material to

Experimentally derived biomimetic chromatographic descriptors for drug-induced phospholipidosis liability prediction

Chrysanthos Stergiopoulos[1] and Klara Valko[2]

[1]*Laboratory of Process Analysis and Design, School of Chemical Engineering, National Technical University of Athens, Iroon Polytechniou 9, 157 75 Zografou, Athens, Greece*
[2]*Bio-Mimetic Chromatography Ltd, Business & Technology Centre, Bessemer Drive, Stevenage, Herts, SG1 2DX, United Kingdom*

ADMET & DMPK **14** (2026) 3460; https://doi.org/10.5599/admet.3460

Table S1. Dataset of compounds used in this study, including CAS registry numbers, biological activity (pEC_{50}) [1,2,3], dataset assignment (training and external test sets) and phospholipidosis (PLD) classification

Compound	CAS	pEC_{50}	Set	PLD classification
Amiloride	2016-88-8	4.85	Train	Weak/moderate inducer
Amiodarone	1951-25-3	5.34	Train	Strong inducer
Amitriptyline	50-48-6	5.09	Test	Strong inducer
Amlodipine	88150-42-9	5.33	Train	Strong inducer
Amoxapine	14028-44-5	5.15	Train	Strong inducer
Atenolol	29122-68-7	3.65	Train	Non-inducer
Atropine	51-55-8	3.91	Test	Non-inducer
Bupropion	34911-55-2	3.6	Train	Non-inducer
Chloroquine	54-05-7	4.67	Train	Weak/moderate inducer
Chlorpromazine	50-53-3	5.19	Train	Strong inducer
Cimetidine	51481-61-9	4.54	Train	Weak/moderate inducer
Citalopram	59729-33-8	4.68	Train	Weak/moderate inducer
Clemastine	15686-51-8	5.28	Test	Strong inducer
Clomipramine	303-49-1	4.88	Test	Weak/moderate inducer
Clozapine	5786-21-0	4.81	Train	Weak/moderate inducer
Desipramine	58-28-6	5.07	Train	Strong inducer
Diclofenac	15307-79-6	3.55	Train	Non-inducer
Disopyramide	3737-09-5	3.56	Test	Non-inducer
Doxycycline	564-25-0	4.49	Train	Weak/moderate inducer
Ebastine	90729-43-4	5.87	Train	Strong inducer
Flecainide	54143-55-4	4.27	Train	Weak/moderate inducer
Fluoxetine	54910-89-3	5.25	Train	Strong inducer
Fluvoxamine	54739-18-3	5.05	Train	Strong inducer
Furosemide	54-31-9	3.7	Train	Non-inducer
Gemfibrozil	25812-30-0	3.7	Train	Non-inducer
Haloperidol	52-86-8	5.00	Test	Weak/moderate inducer
Hydroxyzine	68-88-2	4.40	Train	Weak/moderate inducer
Imipramine	50-49-7	4.68	Train	Weak/moderate inducer
Ketoconazole	65277-42-1	5.26	Train	Strong inducer
Labetalol	32780-64-6	4.73	Test	Weak/moderate inducer
Lidocaine	137-58-6	3.56	Train	Non-inducer
Loperamide	53179-11-6	5.21	Test	Strong inducer
Loratadine	79794-75-5	4.92	Train	Weak/moderate inducer
Loxapine	1977-10-2	4.91	Train	Weak/moderate inducer

Compound	CAS	pEC_{50}	Set	PLD classification
Maprotiline	10262-69-8	5.40	Train	Strong inducer
Menadione	58-27-5	4.72	Train	Weak/moderate inducer
Metergoline	17692-51-2	5.48	Train	Strong inducer
Mexiletine	31828-71-4	4.42	Train	Weak/moderate inducer
Mibefradil	116644-53-2	5.37	Train	Strong inducer
Ofloxacin	82419-36-1	4.30	Train	Weak/moderate inducer
Paracetamol	103-90-2	3.10	Train	Non-inducer
Perphenazine	58-39-9	5.00	Train	Weak/moderate inducer
Procainamide	51-06-9	3.91	Train	Non-inducer
Prochlorperazine	58-38-8	5.51	Train	Strong inducer
Promazine	58-40-2	4.95	Train	Weak/moderate inducer
Promethazine	60-87-7	5.14	Train	Strong inducer
Propafenone	54063-53-5	5.12	Train	Strong inducer
Propranolol	525-66-6	4.85	Train	Weak/moderate inducer
Protriptyline	438-60-8	5.08	Train	Strong inducer
Quinacrine	83-89-6	5.52	Train	Strong inducer
Quinidine	56-54-2	4.84	Train	Weak/moderate inducer
Ribavirin	36791-04-5	3.00	Test	Non-inducer
Risperidone	106266-06-2	4.31	Test	Weak/moderate inducer
Sotalol	3930-20-9	3.63	Train	Non-inducer
Tacrine	321-64-2	4.28	Train	Weak/moderate inducer
Tamoxifen	10540-29-1	5.24	Train	Strong inducer
Thioridazine	50-52-2	5.25	Train	Strong inducer
Tomoxetine	82248-59-7	4.88	Train	Weak/moderate inducer
Trifluoperazine	117-89-5	5.41	Test	Strong inducer
Trimethoprim	738-70-5	3.54	Train	Non-inducer
Valproic acid	99-66-1	3.81	Train	Non-inducer
Vancomycin	1404-90-6	3.63	Train	Non-inducer
Verapamil	52-53-9	4.56	Test	Weak/moderate inducer
Warfarin	81-81-2	3.63	Test	Non-inducer
Zidovudine	30516-87-1	3.10	Train	Non-inducer

Table S2. Biomimetic chromatographic descriptors of the compounds used in this study, including immobilized artificial membrane retention (CHI IAM), human serum albumin binding (log k_{HSA}), and α_1-acid glycoprotein binding (log k_{AGP})

Compound	CHI IAM	log k_{HSA}	log k_{AGP}
Amiloride	31.01	-0.27	-0.13
Amiodarone	62.02	1.59	1.15
Amitriptyline	55.27	0.91	1.05
Amlodipine	43.1	1.11	0.77
Amoxapine	56.46	0.85	1.06
Atenolol	15.75	-1.08	-1.83
Atropine	33.00	-0.33	-0.18
Bupropion	37.73	0.44	0.57
Chloroquine	43.99	0.70	1.12
Chlorpromazine	48.91	1.39	1.01
Cimetidine	15.46	0.02	0.35
Citalopram	48.80	0.40	0.44
Clemastine	60.12	1.25	1.18
Clomipramine	59.61	1.16	1.18
Clozapine	43.92	1.18	0.88
Desipramine	53.1	0.76	0.94
Diclofenac	31.69	1.70	0.13
Disopyramide	24.12	0.21	0.46
Doxycycline	22.50	0.535	-0.41
Ebastine	58.65	1.62	1.34
Flecainide	41.51	-0.05	0.16

CC BY

Compound	CHI IAM	log k_{HSA}	log k_{AGP}
Fluoxetine	54.86	0.99	0.99
Fluvoxamine	50.22	0.4	0.59
Furosemide	20.10	1.24	-1.16
Gemfibrozil	33.66	2.14	0.36
Haloperidol	44.27	0.76	0.73
Hydroxyzine	40.09	0.96	0.84
Imipramine	39.44	0.95	0.83
Ketoconazole	41.13	1.17	0.75
Labetalol	36.15	0.59	0.4
Lidocaine	32.38	-0.71	0.17
Loperamide	45.24	1.04	0.87
Loratadine	44.30	1.38	1.34
Loxapine	50.88	1.05	1.06
Maprotiline	56.73	0.77	0.99
Menadione	30.13	0.78	0.05
Metergoline	55.73	1.28	1.09
Mexiletine	29.52	-1.45	0.16
Mibefradil	55.18	1.10	1.11
Ofloxacin	26.17	-0.4	-0.81
Paracetamol	3.69	0.21	-1.63
Perphenazine	47.64	1.39	1.13
Procainamide	19.30	-0.71	-0.54
Prochlorperazine	60.00	1.37	1.39
Promazine	45.36	1.24	0.92
Promethazine	55.82	0.943	0.97
Propafenone	46.04	0.84	0.92
Propranolol	42.08	0.47	0.84
Protriptyline	51.59	0.69	1.03
Quinacrine	51.02	1.34	1.1
Quinidine	49.78	0.54	0.68
Ribavirin	-10.75	-1.34	-1.70
Risperidone	36.31	0.55	0.63
Sotalol	21.90	-0.75	-1.41
Tacrine	50.13	0.45	0.55
Tamoxifen	52.95	1.57	1.04
Thioridazine	70.24	1.33	1.30
Tomoxetine	50.81	0.81	0.74
Trifluoperazine	68	1.52	1.19
Trimethoprim	12.83	-0.05	-0.07
Valproic acid	35.20	0.85	-0.80
Vancomycin	25.40	-1.15	-1.50
Verapamil	44.82	1.00	0.58
Warfarin	23.53	1.42	0.69
Zidovudine	1.56	-0.875	-1.71

Table S3. Physicochemical and ionization-related descriptors of the studied compounds, including molecular properties (MW, HBD, HBA, TPSA), lipophilicity (log P, log $D_{7.4}$), fractional charge distribution (f_{pos}, f_{neg}, f_{zw}), and Abraham hydrogen bonding descriptors (*A*, *B*), used for model development

Compound	MW	HBD	HBA	TPSA	log *P*	log $D_{7.4}$	f_{pos}	f_{neg}	f_{zw}	*A*	*B*	Charge
Amiloride	229.63	8	8	156.79	0.1	-1.3	0.98	0	0	1.06	1.97	Base
Amiodarone	645.31	0	4	42.68	7.8	6.38	0.96	0	0	0	1.3	Base
Amlodipine	408.87	3	7	99.88	3.17	-1.56	0.99	0	0	0.36	2.19	Base
Amoxapine	313.78	1	4	36.86	3.07	2.72	0.58	0	0	0.16	1.43	Weak base
Atenolol	266.34	4	5	84.58	0.16	-1.89	0.99	0	0	0.69	2	Base
Bupropion	239.74	1	2	29.1	3.37	2.96	0.64	0	0	0.09	1.02	Weak base
Chloroquine	319.87	1	3	28.16	4.63	2.6	1	0	0	0.13	1.29	Base
Chlorpromazine	318.86	0	2	31.78	5.35	3.34	0.99	0	0	0	0.94	Base
Cimetidine	252.34	3	6	114.19	0.4	0.2	0.22	0	0	0.74	1.86	Weak base
Citalopram	324.39	0	3	36.26	3.76	0.99	0.99	0	0	0	1.08	Base
Clozapine	326.82	1	4	30.87	3.32	4.68	0.58	0	0	0.18	1.44	Base
Desipramine	266.38	1	2	15.27	4.9	1.57	1	0	0	0.09	0.91	Base
Diclofenac	296.15	2	3	49.33	4.4	1.23	0	1	0	0.63	0.96	Acid
Doxycycline	444.43	7	10	181.62	-0.02	-2.39	0	0.01	0.99	1.73	3.15	Zwitterion
Ebastine	469.66	0	3	29.54	7.55	6.14	0.97	0	0	0	1.41	Base
Flecainide	414.34	2	5	59.59	3.78	0.32	0.99	0	0	0.41	1.32	Base
Fluoxetine	309.33	1	2	21.26	4.5	2.44	1	0	0	0.13	0.78	Base
Fluvoxamine	318.33	2	4	56.84	3.63	2.32	0.96	0	0	0.23	1.14	Base
Furosemide	330.74	4	7	131.01	2.03	-1.59	0	1	0	1.35	1.45	Acid
Gemfibrozil	250.33	1	3	46.53	4.39	1.9	0	1	0	0.57	0.71	Acid
Hydroxyzine	374.9	1	4	35.94	3.5	1.94	0.78	0	0	0.1	1.89	Weak base
Imipramine	280.41	0	2	4.8	4.28	2.61	0.99	0	0	0	1.15	Base
Ketoconazole	531.43	0	8	69.06	4.34	3.98	0.15	0	0	0	2.22	Weak base
Lidocaine	234.34	1	3	32.34	2.26	2.44	0.78	0	0	0.12	1.21	Base
Loratadine	382.88	0	4	42.43	5.2	4.94	0	0	0	0	1.14	Base
Loxapine	327.81	0	4	28.07	3.04	2.69	0.58	0	0	0	1.49	Weak Base
Maprotiline	277.41	1	1	12.03	4.85	1.48	1	0	0	0.13	0.68	Base
Menadione	172.18	0	2	34.14	2.42	2.42	0	0	0	0	0.79	Neutral
Metergoline	403.52	1	5	46.5	4.66	3.62	0.92	0	0	0.21	1.48	Base
Mexiletine	179.26	2	2	35.25	2.15	0.94	0.96	0	0	0.23	0.9	Base
Mibefradil	495.63	1	6	67.45	4.97	2.37	1	0	0	0.35	1.8	Base
Ofloxacin	361.37	1	7	73.32	-0.39	-2.34	0.05	0.10	0.85	0.57	2.05	Zwitterionic
Paracetamol	151.16	2	3	49.33	0.46	0.23	0	0	0	1.04	0.86	Neutral
Perphenazine	403.97	1	4	55.25	3.69	3.86	0.74	0	0	0.23	1.84	Base
Procainamide	235.32	2	4	58.36	0.88	-0.83	0.99	0	0	0.5	1.45	Base
Prochlorperazine	373.94	0	3	35.02	4.79	4.15	0.85	0	0	0	1.47	Weak base
Promazine	284.42	0	2	31.78	4.55	2.61	0.99	0	0	0	1.01	Base
Promethazine	284.42	0	2	31.78	4.81	2.35	0.99	0	0	0	1.09	Base
Propafenone	341.44	2	4	58.56	3.41	1.26	0.99	0	0	0.29	1.67	Base
Propranolol	259.34	2	3	41.49	3.09	0.89	0.99	0	0	0.17	1.42	Base
Protriptyline	263.38	1	1	12.03	4.91	4.04	0.88	0	0	0.13	0.73	Base
Quinacrine	399.96	1	4	37.39	6.4	3.81	1	0	0	0.13	1.56	Base
Quinidine	324.42	1	4	45.59	2.64	1.06	0.95	0	0	0.23	1.81	Base
Sotalol	272.36	3	5	86.81	0.24	-1.35	0.91	0	0.08	0.74	1.75	Weak base
Tacrine	198.26	2	2	38.91	2.71	0.48	1	0	0	0.23	0.76	Base
Tamoxifen	371.51	0	2	12.47	6.52	5.2	0.96	0	0	0	1.11	Base
Thioridazine	370.58	0	2	57.08	5.9	4.17	0.99	0	0	0	1.13	Base
Tomoxetine	255.16	1	2	23.1	3.7	1.22	1	0	0	0	0.75	Base
Trimethoprim	290.32	4	7	105.51	0.91	0.71	0.47	0	0	0.28	1.62	Base
Valproic acid	144.21	1	2	37.3	2.75	-0.2	0	1	0	0.61	0.45	Acid
Vancomycin	1449.25	21	33	530.49	-2	-5.18	0	0.12	0.88	5.81	10.56	Zwitterion
Zidovudine	267.24	2	9	109.19	0.05	-0.8	0	0.01	0	0.47	1.84	Neutral

CC BY

Table S4. Summary statistics of pEC_{50} values and PLD class distribution for the training and external test sets.

Set	*n*	pEC_{50} mean	pEC_{50} median	pEC_{50} SD	pEC_{50} min	pEC_{50} max	pEC_{50} Range	Contribution in the set, %		
								Non-inducers	Weak/moderate	Strong
Train	52	4.63	4.85	0.706	3.10	5.87	2.77	25.0	38.5	36.5
Test	13	4.51	4.73	0.765	3.00	5.41	2.41	30.8	38.5	30.8

Table S5. Complete MLR models and statistical parameters for phospholipidosis prediction

Model	Equation	R^2	R^2_{adj}	Q^2_{cv}	Q^2_{ext}	$RMSE_{cv}$	RMSEP	*F*	*p*-value	VIF
log $D_{7.4}$	$pEC_{50} = 4.33 + 0.182 \cdot \log D_{7.4}$	0.365	0.353	0.301	0.676	0.584	0.423	28.8	2.08×10^{-6}	1.00
log $D_{7.4}$ + f_{pos}	$pEC_{50} = 3.68 + 0.177 \cdot \log D_{7.4} + 0.878 \cdot f_{pos}$	0.579	0.561	0.479	0.761	0.504	0.364	33.6	6.38×10^{-10}	1.02
log *P*	$pEC_{50} = 3.86 + 0.232 \cdot \log P$	0.478	0.467	0.420	0.648	0.533	0.441	45.8	1.40×10^{-8}	1.00
log *P* + f_{pos}	$pEC_{50} = 3.43 + 0.208 \cdot \log P + 0.681 \cdot f_{pos}$	0.601	0.585	0.487	0.680	0.501	0.421	36.9	1.65×10^{-10}	1.08
CHI IAM	$pEC_{50} = 3.08 + 0.0382 \cdot \text{CHI IAM}$	0.699	0.693	0.657	0.821	0.410	0.315	116	1.21×10^{-14}	1.00
CHI IAM + f_{neg}	$pEC_{50} = 3.23 + 0.0356 \cdot \text{CHI IAM} - 0.347 \cdot f_{neg}$	0.724	0.713	0.689	0.823	0.390	0.313	64.3	1.97×10^{-14}	1.11
CHI IAM + log k_{AGP}	$pEC_{50} = 3.59 + 0.0225 \cdot \text{CHI IAM} + 0.317 \cdot \log k_{AGP}$	0.740	0.729	0.700	0.766	0.383	0.360	69.8	4.61×10^{-15}	3.69
log k_{AGP}	$pEC_{50} = 4.36 + 0.655 \cdot \log k_{AGP}$	0.677	0.671	0.648	0.539	0.415	0.506	105	7.12×10^{-14}	1.00
log k_{AGP} + f_{pos}	$pEC_{50} = 4.06 + 0.594 \cdot \log k_{AGP} + 0.444 \cdot f_{pos}$	0.726	0.715	0.665	0.586	0.404	0.479	64.9	1.71×10^{-14}	1.17
log k_{HSA}	$pEC_{50} = 4.32 + 0.482 \cdot \log k_{HSA}$	0.313	0.299	0.219	0.457	0.618	0.549	22.8	1.63×10^{-5}	1.00
log k_{HSA} + f_{pos}	$pEC_{50} = 3.47 + 0.550 \cdot \log k_{HSA} + 1.08 \cdot f_{pos}$	0.631	0.616	0.513	0.651	0.488	0.440	42.0	2.42×10^{-11}	1.42
log k_{HSA} + f_{pos} + f_{neg}	$pEC_{50} = 3.70 + 0.544 \cdot \log k_{HSA} + 0.888 \cdot f_{pos} - 0.562 \cdot f_{neg}$	0.695	0.676	0.597	0.682	0.444	0.420	36.5	1.97×10^{-12}	1.50

Table S6. Performance of PLS models using CHI IAM and log k_{AGP}

Model	Components	R^2	Q^2_{cv}	Q^2_{ext}	$RMSE_{CV}$	RMSEP
CHI IAM + log k_{AGP}	1	0.748	0.714	0.747	0.369	0.435
CHI IAM + log k_{AGP}	2	0.769	0.700	0.759	0.378	0.428

Table S7. Full performance of all ordinal regression models

Model	log Lik	AIC	BIC	R^2*	Accuracy			Macro F1			Macro OVR		
					Train	CV	Test	Train	CV	Test	Train AUC	CV AUC	Test AUC
CHI IAM	-31.5	69.1	74.9	0.440	0.692	0.692	0.769	0.689	0.687	0.778	0.876	0.860	0.923
CHI IAM + f_{neg}	-30.5	69.0	76.8	0.458	0.731	0.731	0.769	0.734	0.730	0.778	0.882	0.848	0.906
CHI IAM + log k_{AGP}	-30.2	68.4	76.2	0.463	0.692	0.654	0.615	0.692	0.656	0.588	0.886	0.838	0.940
log k_{AGP}	-35.0	76.0	81.9	0.377	0.692	0.673	0.769	0.692	0.670	0.761	0.837	0.823	0.861
log k_{AGP} + f_{pos}	-30.9	69.8	77.6	0.451	0.769	0.692	0.692	0.777	0.697	0.652	0.886	0.867	0.829
log k_{HSA}	-48.7	103	109	0.134	0.558	0.538	0.615	0.555	0.534	0.622	0.723	0.704	0.795
log k_{HSA} + f_{pos}	-37.4	82.9	90.7	0.334	0.654	0.654	0.538	0.653	0.646	0.509	0.823	0.794	0.839
log k_{HSA} + f_{pos} + f_{neg}	-34.1	78.2	88.0	0.394	0.673	0.635	0.615	0.675	0.630	0.613	0.848	0.808	0.871
log $D_{7.4}$	-46.8	99.5	105	0.169	0.519	0.558	0.692	0.522	0.555	0.689	0.741	0.721	0.915
log $D_{7.4}$ + f_{pos}	-37.9	83.7	91.5	0.327	0.596	0.596	0.615	0.599	0.596	0.613	0.828	0.801	0.898
log *P* + f_{pos}	-36.1	80.2	88.0	0.358	0.596	0.596	0.692	0.598	0.590	0.689	0.842	0.806	0.855
log *P*	-41.5	89.0	94.9	0.262	0.654	0.654	0.615	0.644	0.644	0.588	0.796	0.764	0.847

*McFadden's R^2

Table S8. Coefficients of ordinal regression models

Model	Term	Ordinal logit coefficient (β)	Std. Error	*z*-value	*p*-value	95% CI low	95% CI high
CHI IAM	CHI IAM	0.198	0.0427	4.64	3.50×10^{-6}	0.114	0.282
CHI IAM + f_{neg}	CHI IAM	0.188	0.0432	4.35	1.37×10^{-5}	0.103	0.272
	f_{neg}	-1.63	1.15	-1.42	0.156	-3.88	0.624
CHI IAM + log k_{AGP}	CHI IAM	0.143	0.0517	2.77	0.00567	0.0417	0.244
	log k_{AGP}	1.38	0.885	1.56	0.119	-0.354	3.11
log k_{AGP}	log k_{AGP}	3.15	0.742	4.24	2.19×10^{-5}	1.70	4.61
log k_{AGP} + f_{pos}	log k_{AGP}	3.33	0.845	3.95	7.94×10^{-5}	1.68	4.99
	f_{pos}	2.99	1.14	2.63	0.00866	0.757	5.22
log k_{HSA}	log k_{HSA}	1.43	0.408	3.51	4.51×10^{-4}	0.631	2.23

Model	Term	Ordinal logit coefficient (β)	Std. Error	*z*-value	*p*-value	95% CI low	95% CI high
log k_{HSA} + f_{pos}	log k_{HSA}	2.12	0.506	4.19	2.74×10^{-5}	1.13	3.11
	f_{pos}	4.59	1.16	3.95	7.90×10^{-5}	2.31	6.87
log k_{HSA} + f_{pos} + f_{neg}	log k_{HSA}	2.32	0.568	4.08	4.53×10^{-5}	1.20	3.43
	f_{pos}	3.99	1.17	3.42	6.30×10^{-4}	1.70	6.28
	f_{neg}	-2.85	1.23	-2.32	0.0203	-5.26	-0.444
log $D_{7.4}$	log $D_{7.4}$	0.569	0.150	3.79	1.53×10^{-4}	0.275	0.864
log $D_{7.4}$ + f_{pos}	log $D_{7.4}$	0.718	0.179	4.01	6.00×10^{-5}	0.367	1.07
	f_{pos}	3.64	1.00	3.64	2.75×10^{-4}	1.68	5.61
log *P* + f_{pos}	log *P*	0.900	0.212	4.24	2.20×10^{-5}	0.484	1.32
	f_{pos}	2.96	0.983	3.01	0.00259	1.03	4.89
log *P*	log *P*	0.866	0.196	4.42	1.00×10^{-5}	0.482	1.25

Table S9. Cutpoint estimates of ordinal regression models

Model	Cutpoint	Estimate	Std. Error	*z*-value	*p*-value	95% CI low	95% CI high
CHI IAM	0/1	5.69	1.47	3.88	1.05×10^{-4}	2.81	8.56
	1/2	1.36	0.232	5.87	4.36×10^{-9}	0.908	1.82
CHI IAM + f_{neg}	0/1	4.79	1.50	3.19	0.00141	1.85	7.73
	1/2	1.46	0.245	5.93	2.95×10^{-9}	0.975	1.94
CHI IAM + log k_{AGP}	0/1	3.88	1.72	2.25	0.0244	0.500	7.25
	1/2	1.45	0.245	5.92	3.27×10^{-9}	0.969	1.93
log k_{AGP}	0/1	-0.568	0.536	-1.06	0.289	-1.62	0.482
	1/2	1.21	0.226	5.37	7.98×10^{-8}	0.770	1.66
log k_{AGP} + f_{pos}	0/1	1.37	0.924	1.48	0.139	-0.443	3.18
	1/2	1.42	0.247	5.73	1.00×10^{-8}	0.933	1.90
log k_{HSA}	0/1	-0.517	0.389	-1.33	0.184	-1.28	0.246
	1/2	0.748	0.206	3.64	2.74×10^{-4}	0.345	1.15
log k_{HSA} + f_{pos}	0/1	2.85	0.946	3.01	0.00262	0.993	4.70
	1/2	1.15	0.224	5.13	2.90×10^{-7}	0.711	1.59
log k_{HSA} + f_{pos} + f_{neg}	0/1	1.84	0.965	1.90	0.0571	-0.055	3.73
	1/2	1.30	0.236	5.53	3.24×10^{-8}	0.842	1.77
log $D_{7.4}$	0/1	-0.502	0.386	-1.30	0.193	-1.26	0.254
	1/2	0.791	0.204	3.87	1.08×10^{-4}	0.390	1.19
log $D_{7.4}$ + f_{pos}	0/1	2.05	0.852	2.41	0.0161	0.381	3.72
	1/2	1.07	0.214	5.02	5.11×10^{-7}	0.655	1.49
log *P* + f_{pos}	0/1	3.15	0.985	3.20	0.00140	1.22	5.08
	1/2	1.16	0.218	5.31	1.08×10^{-7}	0.731	1.58
log *P*	0/1	1.12	0.610	1.83	0.0677	-0.0810	2.31
	1/2	0.961	0.210	4.58	4.57×10^{-6}	0.550	1.37

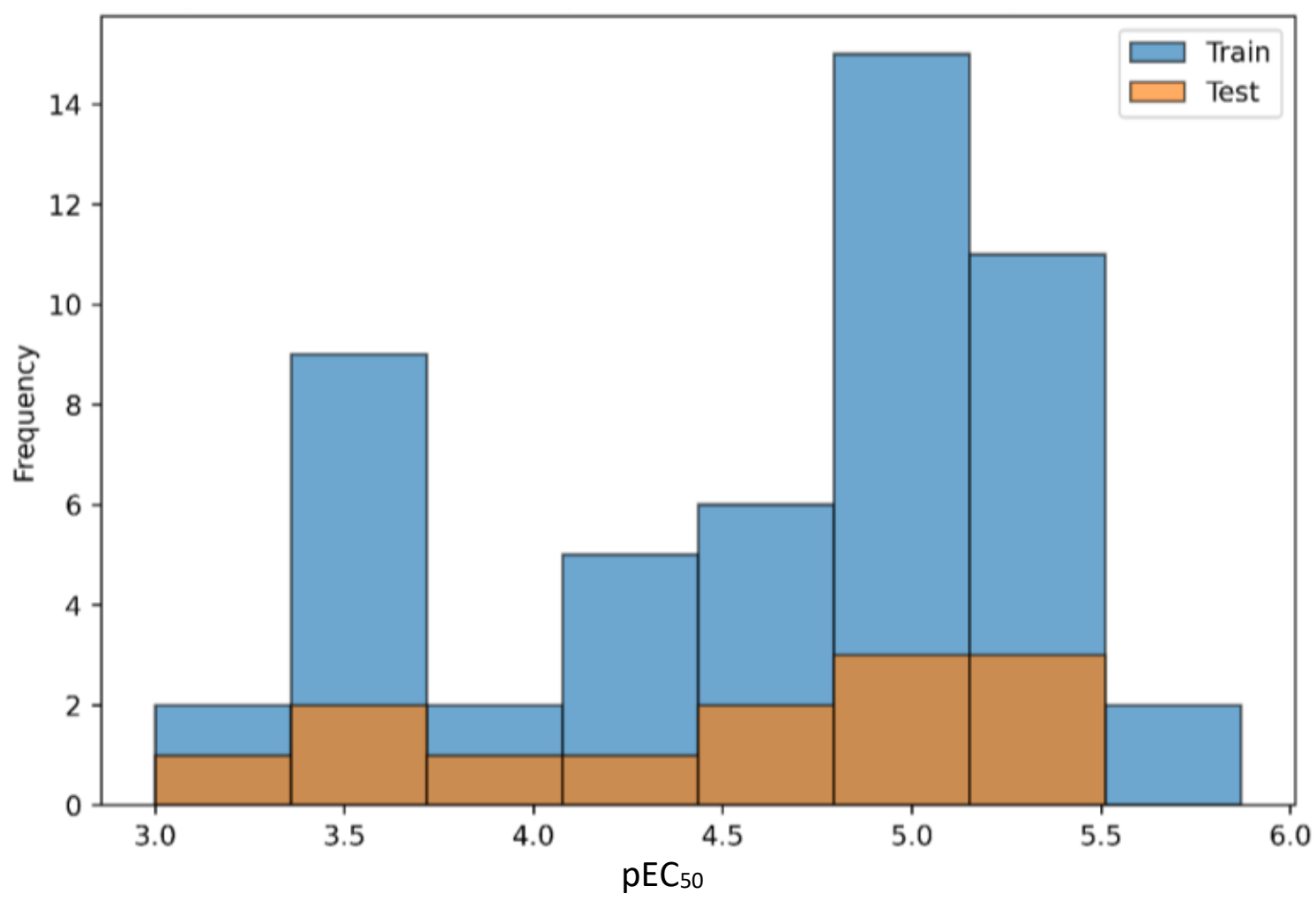

Figure S1. Distribution of pEC_{50} values in the training and external test sets, demonstrating the preservation of activity range and representativeness between the two subsets

(cc) BY

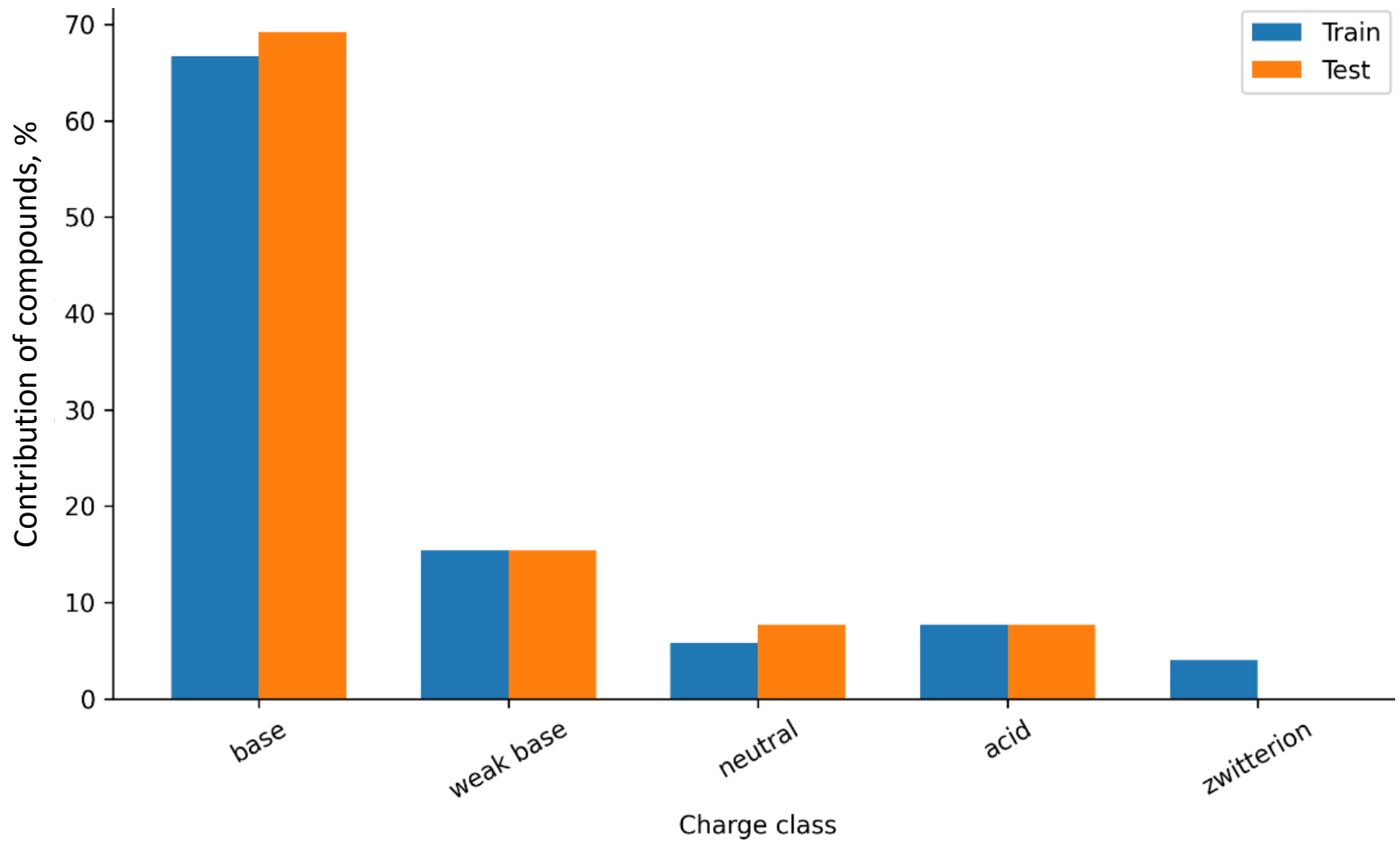

Figure S2. Distribution of compounds across charge classes in the training and test sets, showing consistent representation of ionization states following ordinal dataset splitting

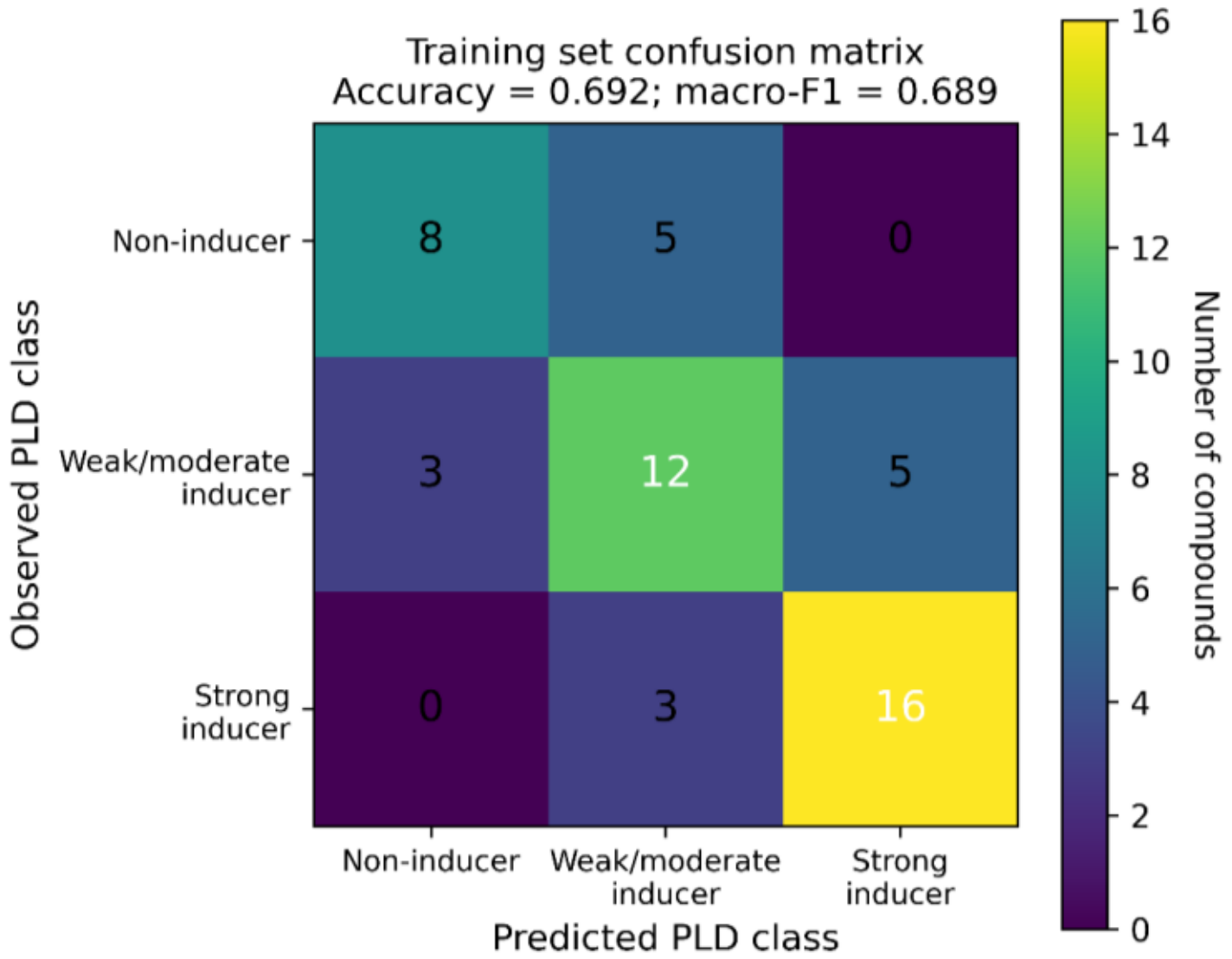

Figure S3. Training-set confusion matrix for the ordinal regression model based on CHI IAM. Misclassifications occurred mainly between adjacent phospholipidosis classes, with no direct confusion between non-inducers and strong inducers

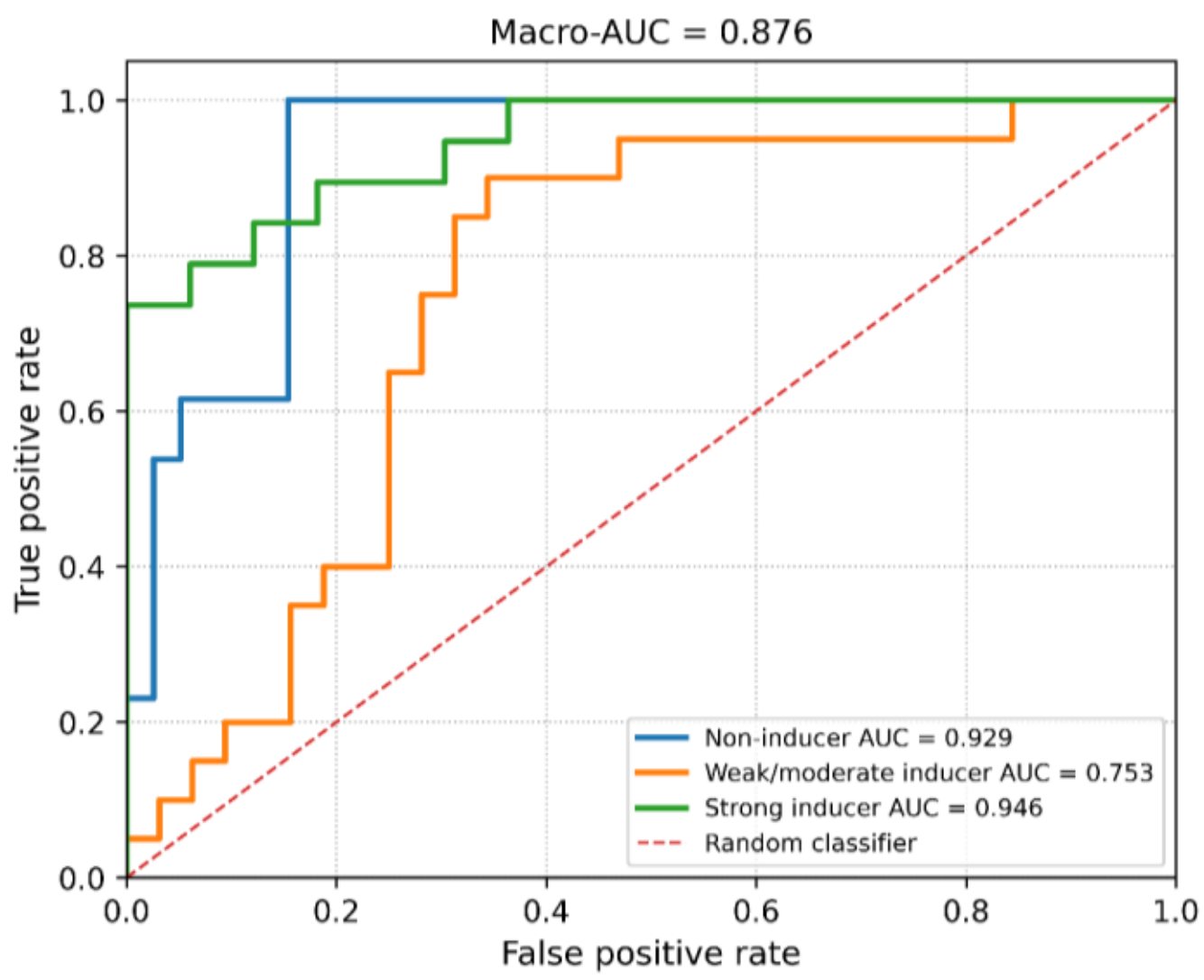

Figure S4. Training-set multiclass receiver operating characteristic curves for the CHI IAM ordinal regression model. Curves were generated using a one-vs-rest approach, in which each PLD class was treated as the positive class, and the remaining two classes were combined as the negative group

References

[1] L. LeCureux, C.S. Cheng, J. Herbst, et al. Evaluation and validation of multiple cell lines and primary mouse macrophages to predict phospholipidosis potential. *Toxicology in Vitro* **25** (2011) 1934-1943. https://dx.doi.org/10.1016/j.tiv.2011.06.017

[2] S.A. Shahane, R. Huang, D. Gerhold, et al. Detection of phospholipidosis induction: a cell-based assay in high-throughput and high-content format. *SLAS Discovery* **19** (2014) 66-76. https://dx.doi.org/10.1177/1087057113502851

[3] F. Atienzar, H. Gerets, S. Dufrane, et al. Determination of phospholipidosis potential based on gene expression analysis in HepG2 cells. *Toxicological Sciences* **96** (2007) 101-114. https://dx.doi.org/10.1093/toxsci/kfl184

(cc) BY
