# Supplementary material for: Experimentally derived biomimetic chromatographic descriptors for drug-induced phospholipidosis liability prediction
Source: ADMET DMPK. 2026 Jul 10;14:3460. doi: 10.5599/admet.3460 (PMC13499669; doi:10.5599/admet.3460)
Supplement: Supplementary file 2 [file ADMET-14-3460-S1.docx]

ADMET & DMPK **14** (2026) S3460

**Open Access : ISSN : 1848-7718**
[**http://www.pub.iapchem.org/ojs/index.php/admet**](http://www.pub.iapchem.org/ojs/index.php/admet)

Supplementary material to

Experimentally derived biomimetic chromatographic descriptors for drug-induced phospholipidosis liability prediction

Chrysanthos Stergiopoulos^1^^<https://orcid.org/0000-0001-6002-4870>^ and Klara Valko^2^^<https://orcid.org/0000-0003-4605-2941>^

^1^Laboratory of Process Analysis and Design, School of Chemical Engineering, National Technical University of Athens, Iroon Polytechniou 9, 157 75 Zografou, Athens, Greece
^2^Bio-Mimetic Chromatography Ltd, Business & Technology Centre, Bessemer Drive, Stevenage, Herts, SG1 2DX, United Kingdom

ADMET & DMPK **14** (2026) 3460; <https://doi.org/10.5599/admet.3460>

**Table S1.** Dataset of compounds used in this study, including CAS registry numbers, biological activity (pEC₅₀) [1,2,3], dataset assignment (training and external test sets) and phospholipidosis (PLD) classification

| Compound | CAS | pEC_50_ | Set | PLD classification |
| --- | --- | --- | --- | --- |
| Amiloride | 2016-88-8 | 4.85 | Train | Weak/moderate inducer |
| Amiodarone | 1951-25-3 | 5.34 | Train | Strong inducer |
| Amitriptyline | 50-48-6 | 5.09 | Test | Strong inducer |
| Amlodipine | 88150-42-9 | 5.33 | Train | Strong inducer |
| Amoxapine | 14028-44-5 | 5.15 | Train | Strong inducer |
| Atenolol | 29122-68-7 | 3.65 | Train | Non-inducer |
| Atropine | 51-55-8 | 3.91 | Test | Non-inducer |
| Bupropion | 34911-55-2 | 3.6 | Train | Non-inducer |
| Chloroquine | 54-05-7 | 4.67 | Train | Weak/moderate inducer |
| Chlorpromazine | 50-53-3 | 5.19 | Train | Strong inducer |
| Cimetidine | 51481-61-9 | 4.54 | Train | Weak/moderate inducer |
| Citalopram | 59729-33-8 | 4.68 | Train | Weak/moderate inducer |
| Clemastine | 15686-51-8 | 5.28 | Test | Strong inducer |
| Clomipramine | 303-49-1 | 4.88 | Test | Weak/moderate inducer |
| Clozapine | 5786-21-0 | 4.81 | Train | Weak/moderate inducer |
| Desipramine | 58-28-6 | 5.07 | Train | Strong inducer |
| Diclofenac | 15307-79-6 | 3.55 | Train | Non-inducer |
| Disopyramide | 3737-09-5 | 3.56 | Test | Non-inducer |
| Doxycycline | 564-25-0 | 4.49 | Train | Weak/moderate inducer |
| Ebastine | 90729-43-4 | 5.87 | Train | Strong inducer |
| Flecainide | 54143-55-4 | 4.27 | Train | Weak/moderate inducer |
| Fluoxetine | 54910-89-3 | 5.25 | Train | Strong inducer |
| Fluvoxamine | 54739-18-3 | 5.05 | Train | Strong inducer |
| Furosemide | 54-31-9 | 3.7 | Train | Non-inducer |
| Gemfibrozil | 25812-30-0 | 3.7 | Train | Non-inducer |
| Haloperidol | 52-86-8 | 5.00 | Test | Weak/moderate inducer |
| Hydroxyzine | 68-88-2 | 4.40 | Train | Weak/moderate inducer |
| Imipramine | 50-49-7 | 4.68 | Train | Weak/moderate inducer |
| Ketoconazole | 65277-42-1 | 5.26 | Train | Strong inducer |
| Labetalol | 32780-64-6 | 4.73 | Test | Weak/moderate inducer |
| Lidocaine | 137-58-6 | 3.56 | Train | Non-inducer |
| Loperamide | 53179-11-6 | 5.21 | Test | Strong inducer |
| Loratadine | 79794-75-5 | 4.92 | Train | Weak/moderate inducer |
| Loxapine | 1977-10-2 | 4.91 | Train | Weak/moderate inducer |
| Maprotiline | 10262-69-8 | 5.40 | Train | Strong inducer |
| Menadione | 58-27-5 | 4.72 | Train | Weak/moderate inducer |
| Metergoline | 17692-51-2 | 5.48 | Train | Strong inducer |
| Mexiletine | 31828-71-4 | 4.42 | Train | Weak/moderate inducer |
| Mibefradil | 116644-53-2 | 5.37 | Train | Strong inducer |
| Ofloxacin | 82419-36-1 | 4.30 | Train | Weak/moderate inducer |
| Paracetamol | 103-90-2 | 3.10 | Train | Non-inducer |
| Perphenazine | 58-39-9 | 5.00 | Train | Weak/moderate inducer |
| Procainamide | 51-06-9 | 3.91 | Train | Non-inducer |
| Prochlorperazine | 58-38-8 | 5.51 | Train | Strong inducer |
| Promazine | 58-40-2 | 4.95 | Train | Weak/moderate inducer |
| Promethazine | 60-87-7 | 5.14 | Train | Strong inducer |
| Propafenone | 54063-53-5 | 5.12 | Train | Strong inducer |
| Propranolol | 525-66-6 | 4.85 | Train | Weak/moderate inducer |
| Protriptyline | 438-60-8 | 5.08 | Train | Strong inducer |
| Quinacrine | 83-89-6 | 5.52 | Train | Strong inducer |
| Quinidine | 56-54-2 | 4.84 | Train | Weak/moderate inducer |
| Ribavirin | 36791-04-5 | 3.00 | Test | Non-inducer |
| Risperidone | 106266-06-2 | 4.31 | Test | Weak/moderate inducer |
| Sotalol | 3930-20-9 | 3.63 | Train | Non-inducer |
| Tacrine | 321-64-2 | 4.28 | Train | Weak/moderate inducer |
| Tamoxifen | 10540-29-1 | 5.24 | Train | Strong inducer |
| Thioridazine | 50-52-2 | 5.25 | Train | Strong inducer |
| Tomoxetine | 82248-59-7 | 4.88 | Train | Weak/moderate inducer |
| Trifluoperazine | 117-89-5 | 5.41 | Test | Strong inducer |
| Trimethoprim | 738-70-5 | 3.54 | Train | Non-inducer |
| Valproic acid | 99-66-1 | 3.81 | Train | Non-inducer |
| Vancomycin | 1404-90-6 | 3.63 | Train | Non-inducer |
| Verapamil | 52-53-9 | 4.56 | Test | Weak/moderate inducer |
| Warfarin | 81-81-2 | 3.63 | Test | Non-inducer |
| Zidovudine | 30516-87-1 | 3.10 | Train | Non-inducer |

**Table S2.** Biomimetic chromatographic descriptors of the compounds used in this study, including immobilized artificial membrane retention (CHI IAM), human serum albumin binding (log *k*_HSA_), and α₁-acid glycoprotein binding (log *k*_AGP_)

| Compound | CHI IAM | log *k*_HSA_ | log *k*_AGP_ |
| --- | --- | --- | --- |
| Amiloride | 31.01 | -0.27 | -0.13 |
| Amiodarone | 62.02 | 1.59 | 1.15 |
| Amitriptyline | 55.27 | 0.91 | 1.05 |
| Amlodipine | 43.1 | 1.11 | 0.77 |
| Amoxapine | 56.46 | 0.85 | 1.06 |
| Atenolol | 15.75 | -1.08 | -1.83 |
| Atropine | 33.00 | -0.33 | -0.18 |
| Bupropion | 37.73 | 0.44 | 0.57 |
| Chloroquine | 43.99 | 0.70 | 1.12 |
| Chlorpromazine | 48.91 | 1.39 | 1.01 |
| Cimetidine | 15.46 | 0.02 | 0.35 |
| Citalopram | 48.80 | 0.40 | 0.44 |
| Clemastine | 60.12 | 1.25 | 1.18 |
| Clomipramine | 59.61 | 1.16 | 1.18 |
| Clozapine | 43.92 | 1.18 | 0.88 |
| Desipramine | 53.1 | 0.76 | 0.94 |
| Diclofenac | 31.69 | 1.70 | 0.13 |
| Disopyramide | 24.12 | 0.21 | 0.46 |
| Doxycycline | 22.50 | 0.535 | -0.41 |
| Ebastine | 58.65 | 1.62 | 1.34 |
| Flecainide | 41.51 | -0.05 | 0.16 |
| Fluoxetine | 54.86 | 0.99 | 0.99 |
| Fluvoxamine | 50.22 | 0.4 | 0.59 |
| Furosemide | 20.10 | 1.24 | -1.16 |
| Gemfibrozil | 33.66 | 2.14 | 0.36 |
| Haloperidol | 44.27 | 0.76 | 0.73 |
| Hydroxyzine | 40.09 | 0.96 | 0.84 |
| Imipramine | 39.44 | 0.95 | 0.83 |
| Ketoconazole | 41.13 | 1.17 | 0.75 |
| Labetalol | 36.15 | 0.59 | 0.4 |
| Lidocaine | 32.38 | -0.71 | 0.17 |
| Loperamide | 45.24 | 1.04 | 0.87 |
| Loratadine | 44.30 | 1.38 | 1.34 |
| Loxapine | 50.88 | 1.05 | 1.06 |
| Maprotiline | 56.73 | 0.77 | 0.99 |
| Menadione | 30.13 | 0.78 | 0.05 |
| Metergoline | 55.73 | 1.28 | 1.09 |
| Mexiletine | 29.52 | -1.45 | 0.16 |
| Mibefradil | 55.18 | 1.10 | 1.11 |
| Ofloxacin | 26.17 | -0.4 | -0.81 |
| Paracetamol | 3.69 | 0.21 | -1.63 |
| Perphenazine | 47.64 | 1.39 | 1.13 |
| Procainamide | 19.30 | -0.71 | -0.54 |
| Prochlorperazine | 60.00 | 1.37 | 1.39 |
| Promazine | 45.36 | 1.24 | 0.92 |
| Promethazine | 55.82 | 0.943 | 0.97 |
| Propafenone | 46.04 | 0.84 | 0.92 |
| Propranolol | 42.08 | 0.47 | 0.84 |
| Protriptyline | 51.59 | 0.69 | 1.03 |
| Quinacrine | 51.02 | 1.34 | 1.1 |
| Quinidine | 49.78 | 0.54 | 0.68 |
| Ribavirin | -10.75 | -1.34 | -1.70 |
| Risperidone | 36.31 | 0.55 | 0.63 |
| Sotalol | 21.90 | -0.75 | -1.41 |
| Tacrine | 50.13 | 0.45 | 0.55 |
| Tamoxifen | 52.95 | 1.57 | 1.04 |
| Thioridazine | 70.24 | 1.33 | 1.30 |
| Tomoxetine | 50.81 | 0.81 | 0.74 |
| Trifluoperazine | 68 | 1.52 | 1.19 |
| Trimethoprim | 12.83 | -0.05 | -0.07 |
| Valproic acid | 35.20 | 0.85 | -0.80 |
| Vancomycin | 25.40 | -1.15 | -1.50 |
| Verapamil | 44.82 | 1.00 | 0.58 |
| Warfarin | 23.53 | 1.42 | 0.69 |
| Zidovudine | 1.56 | -0.875 | -1.71 |

**Table S3.** Physicochemical and ionization-related descriptors of the studied compounds, including molecular properties (MW, HBD, HBA, TPSA), lipophilicity (log P, log D_7.4_), fractional charge distribution (*f*_pos_, *f*_neg_, *f*_zw_), and Abraham hydrogen bonding descriptors (*A*, *B*), used for model development

| Compound | MW | HBD | HBA | TPSA | log *P* | log *D*_7.4_ | *f*_pos_ | *f*_neg_ | *f*_zw_ | *A* | *B* | Charge |
| --- | --- | --- | --- | --- | --- | --- | --- | --- | --- | --- | --- | --- |
| Amiloride | 229.63 | 8 | 8 | 156.79 | 0.1 | -1.3 | 0.98 | 0 | 0 | 1.06 | 1.97 | Base |
| Amiodarone | 645.31 | 0 | 4 | 42.68 | 7.8 | 6.38 | 0.96 | 0 | 0 | 0 | 1.3 | Base |
| Amlodipine | 408.87 | 3 | 7 | 99.88 | 3.17 | -1.56 | 0.99 | 0 | 0 | 0.36 | 2.19 | Base |
| Amoxapine | 313.78 | 1 | 4 | 36.86 | 3.07 | 2.72 | 0.58 | 0 | 0 | 0.16 | 1.43 | Weak base |
| Atenolol | 266.34 | 4 | 5 | 84.58 | 0.16 | -1.89 | 0.99 | 0 | 0 | 0.69 | 2 | Base |
| Bupropion | 239.74 | 1 | 2 | 29.1 | 3.37 | 2.96 | 0.64 | 0 | 0 | 0.09 | 1.02 | Weak base |
| Chloroquine | 319.87 | 1 | 3 | 28.16 | 4.63 | 2.6 | 1 | 0 | 0 | 0.13 | 1.29 | Base |
| Chlorpromazine | 318.86 | 0 | 2 | 31.78 | 5.35 | 3.34 | 0.99 | 0 | 0 | 0 | 0.94 | Base |
| Cimetidine | 252.34 | 3 | 6 | 114.19 | 0.4 | 0.2 | 0.22 | 0 | 0 | 0.74 | 1.86 | Weak base |
| Citalopram | 324.39 | 0 | 3 | 36.26 | 3.76 | 0.99 | 0.99 | 0 | 0 | 0 | 1.08 | Base |
| Clozapine | 326.82 | 1 | 4 | 30.87 | 3.32 | 4.68 | 0.58 | 0 | 0 | 0.18 | 1.44 | Base |
| Desipramine | 266.38 | 1 | 2 | 15.27 | 4.9 | 1.57 | 1 | 0 | 0 | 0.09 | 0.91 | Base |
| Diclofenac | 296.15 | 2 | 3 | 49.33 | 4.4 | 1.23 | 0 | 1 | 0 | 0.63 | 0.96 | Acid |
| Doxycycline | 444.43 | 7 | 10 | 181.62 | -0.02 | -2.39 | 0 | 0.01 | 0.99 | 1.73 | 3.15 | Zwitterion |
| Ebastine | 469.66 | 0 | 3 | 29.54 | 7.55 | 6.14 | 0.97 | 0 | 0 | 0 | 1.41 | Base |
| Flecainide | 414.34 | 2 | 5 | 59.59 | 3.78 | 0.32 | 0.99 | 0 | 0 | 0.41 | 1.32 | Base |
| Fluoxetine | 309.33 | 1 | 2 | 21.26 | 4.5 | 2.44 | 1 | 0 | 0 | 0.13 | 0.78 | Base |
| Fluvoxamine | 318.33 | 2 | 4 | 56.84 | 3.63 | 2.32 | 0.96 | 0 | 0 | 0.23 | 1.14 | Base |
| Furosemide | 330.74 | 4 | 7 | 131.01 | 2.03 | -1.59 | 0 | 1 | 0 | 1.35 | 1.45 | Acid |
| Gemfibrozil | 250.33 | 1 | 3 | 46.53 | 4.39 | 1.9 | 0 | 1 | 0 | 0.57 | 0.71 | Acid |
| Hydroxyzine | 374.9 | 1 | 4 | 35.94 | 3.5 | 1.94 | 0.78 | 0 | 0 | 0.1 | 1.89 | Weak base |
| Imipramine | 280.41 | 0 | 2 | 4.8 | 4.28 | 2.61 | 0.99 | 0 | 0 | 0 | 1.15 | Base |
| Ketoconazole | 531.43 | 0 | 8 | 69.06 | 4.34 | 3.98 | 0.15 | 0 | 0 | 0 | 2.22 | Weak base |
| Lidocaine | 234.34 | 1 | 3 | 32.34 | 2.26 | 2.44 | 0.78 | 0 | 0 | 0.12 | 1.21 | Base |
| Loratadine | 382.88 | 0 | 4 | 42.43 | 5.2 | 4.94 | 0 | 0 | 0 | 0 | 1.14 | Base |
| Loxapine | 327.81 | 0 | 4 | 28.07 | 3.04 | 2.69 | 0.58 | 0 | 0 | 0 | 1.49 | Weak Base |
| Maprotiline | 277.41 | 1 | 1 | 12.03 | 4.85 | 1.48 | 1 | 0 | 0 | 0.13 | 0.68 | Base |
| Menadione | 172.18 | 0 | 2 | 34.14 | 2.42 | 2.42 | 0 | 0 | 0 | 0 | 0.79 | Neutral |
| Metergoline | 403.52 | 1 | 5 | 46.5 | 4.66 | 3.62 | 0.92 | 0 | 0 | 0.21 | 1.48 | Base |
| Mexiletine | 179.26 | 2 | 2 | 35.25 | 2.15 | 0.94 | 0.96 | 0 | 0 | 0.23 | 0.9 | Base |
| Mibefradil | 495.63 | 1 | 6 | 67.45 | 4.97 | 2.37 | 1 | 0 | 0 | 0.35 | 1.8 | Base |
| Ofloxacin | 361.37 | 1 | 7 | 73.32 | -0.39 | -2.34 | 0.05 | 0.10 | 0.85 | 0.57 | 2.05 | Zwitterionic |
| Paracetamol | 151.16 | 2 | 3 | 49.33 | 0.46 | 0.23 | 0 | 0 | 0 | 1.04 | 0.86 | Neutral |
| Perphenazine | 403.97 | 1 | 4 | 55.25 | 3.69 | 3.86 | 0.74 | 0 | 0 | 0.23 | 1.84 | Base |
| Procainamide | 235.32 | 2 | 4 | 58.36 | 0.88 | -0.83 | 0.99 | 0 | 0 | 0.5 | 1.45 | Base |
| Prochlorperazine | 373.94 | 0 | 3 | 35.02 | 4.79 | 4.15 | 0.85 | 0 | 0 | 0 | 1.47 | Weak base |
| Promazine | 284.42 | 0 | 2 | 31.78 | 4.55 | 2.61 | 0.99 | 0 | 0 | 0 | 1.01 | Base |
| Promethazine | 284.42 | 0 | 2 | 31.78 | 4.81 | 2.35 | 0.99 | 0 | 0 | 0 | 1.09 | Base |
| Propafenone | 341.44 | 2 | 4 | 58.56 | 3.41 | 1.26 | 0.99 | 0 | 0 | 0.29 | 1.67 | Base |
| Propranolol | 259.34 | 2 | 3 | 41.49 | 3.09 | 0.89 | 0.99 | 0 | 0 | 0.17 | 1.42 | Base |
| Protriptyline | 263.38 | 1 | 1 | 12.03 | 4.91 | 4.04 | 0.88 | 0 | 0 | 0.13 | 0.73 | Base |
| Quinacrine | 399.96 | 1 | 4 | 37.39 | 6.4 | 3.81 | 1 | 0 | 0 | 0.13 | 1.56 | Base |
| Quinidine | 324.42 | 1 | 4 | 45.59 | 2.64 | 1.06 | 0.95 | 0 | 0 | 0.23 | 1.81 | Base |
| Sotalol | 272.36 | 3 | 5 | 86.81 | 0.24 | -1.35 | 0.91 | 0 | 0.08 | 0.74 | 1.75 | Weak base |
| Tacrine | 198.26 | 2 | 2 | 38.91 | 2.71 | 0.48 | 1 | 0 | 0 | 0.23 | 0.76 | Base |
| Tamoxifen | 371.51 | 0 | 2 | 12.47 | 6.52 | 5.2 | 0.96 | 0 | 0 | 0 | 1.11 | Base |
| Thioridazine | 370.58 | 0 | 2 | 57.08 | 5.9 | 4.17 | 0.99 | 0 | 0 | 0 | 1.13 | Base |
| Tomoxetine | 255.16 | 1 | 2 | 23.1 | 3.7 | 1.22 | 1 | 0 | 0 | 0 | 0.75 | Base |
| Trimethoprim | 290.32 | 4 | 7 | 105.51 | 0.91 | 0.71 | 0.47 | 0 | 0 | 0.28 | 1.62 | Base |
| Valproic acid | 144.21 | 1 | 2 | 37.3 | 2.75 | -0.2 | 0 | 1 | 0 | 0.61 | 0.45 | Acid |
| Vancomycin | 1449.25 | 21 | 33 | 530.49 | -2 | -5.18 | 0 | 0.12 | 0.88 | 5.81 | 10.56 | Zwitterion |
| Zidovudine | 267.24 | 2 | 9 | 109.19 | 0.05 | -0.8 | 0 | 0.01 | 0 | 0.47 | 1.84 | Neutral |

**Table S4.** Summary statistics of pEC₅₀ values and PLD class distribution for the training and external test sets.

| Set | *n* | pEC_50_ mean | pEC_50_ median | pEC_50_ SD | pEC_50_ min | pEC_50_ max | pEC_50_ Range | Contribution in the set, % | | |
| --- | --- | --- | --- | --- | --- | --- | --- | --- | --- | --- |
|  |  |  |  |  |  |  |  | Non-inducers | Weak/moderate | Strong |
| Train | 52 | 4.63 | 4.85 | 0.706 | 3.10 | 5.87 | 2.77 | 25.0 | 38.5 | 36.5 |
| Test | 13 | 4.51 | 4.73 | 0.765 | 3.00 | 5.41 | 2.41 | 30.8 | 38.5 | 30.8 |

**Table S5.** Complete MLR models and statistical parameters for phospholipidosis prediction

| Model | Equation | *R*² | *R*²_adj_ | *Q*²_cv_ | *Q*²_ext_ | RMSE_cv_ | RMSEP | *F* | *p*-value | VIF |
| --- | --- | --- | --- | --- | --- | --- | --- | --- | --- | --- |
| log *D*_7.4_ | pEC₅₀ = 4.33 + 0.182·log *D*_7.4_ | 0.365 | 0.353 | 0.301 | 0.676 | 0.584 | 0.423 | 28.8 | 2.08×10^-6^ | 1.00 |
| log *D*_7.4_ + *f*_pos_ | pEC₅₀ = 3.68 + 0.177·log *D*_7.4_ + 0.878·*f*_pos_ | 0.579 | 0.561 | 0.479 | 0.761 | 0.504 | 0.364 | 33.6 | 6.38×10^-10^ | 1.02 |
| log *P* | pEC₅₀ = 3.86 + 0.232·log P | 0.478 | 0.467 | 0.420 | 0.648 | 0.533 | 0.441 | 45.8 | 1.40×10^-8^ | 1.00 |
| log *P* + *f*_pos_ | pEC₅₀ = 3.43 + 0.208·log *P* + 0.681·*f*_pos_ | 0.601 | 0.585 | 0.487 | 0.680 | 0.501 | 0.421 | 36.9 | 1.65×10^-10^ | 1.08 |
| CHI IAM | pEC₅₀ = 3.08 + 0.0382·CHI IAM | 0.699 | 0.693 | 0.657 | 0.821 | 0.410 | 0.315 | 116 | 1.21×10^-14^ | 1.00 |
| CHI IAM  + *f*_neg_ | pEC₅₀ = 3.23 + 0.0356·CHI IAM − 0.347·*f*_neg_ | 0.724 | 0.713 | 0.689 | 0.823 | 0.390 | 0.313 | 64.3 | 1.97×10^-14^ | 1.11 |
| CHI IAM  + log *k*_AGP_ | pEC₅₀ = 3.59 + 0.0225·CHI IAM + 0.317·log *k*_AGP_ | 0.740 | 0.729 | 0.700 | 0.766 | 0.383 | 0.360 | 69.8 | 4.61×10^-15^ | 3.69 |
| log *k*_AGP_ | pEC₅₀ = 4.36 + 0.655·log *k*_AGP_ | 0.677 | 0.671 | 0.648 | 0.539 | 0.415 | 0.506 | 105 | 7.12×10^-14^ | 1.00 |
| log *k*_AGP_  + *f*_pos_ | pEC₅₀ = 4.06 + 0.594·log k_AGP_ + 0.444·*f*_pos_ | 0.726 | 0.715 | 0.665 | 0.586 | 0.404 | 0.479 | 64.9 | 1.71×10^-14^ | 1.17 |
| log *k*_HSA_ | pEC₅₀ = 4.32 + 0.482·log *k*_HSA_ | 0.313 | 0.299 | 0.219 | 0.457 | 0.618 | 0.549 | 22.8 | 1.63×10^-5^ | 1.00 |
| log *k*_HSA_  + *f*_pos_ | pEC₅₀ = 3.47 + 0.550·log *k*_HSA_ + 1.08·*f*_pos_ | 0.631 | 0.616 | 0.513 | 0.651 | 0.488 | 0.440 | 42.0 | 2.42×10^-11^ | 1.42 |
| log *k*_HSA_  + *f*_pos_ + *f*_neg_ | pEC₅₀ = 3.70+0.544·log *k*_HSA_+0.888·*f*_pos_-0.562·*f*_neg_ | 0.695 | 0.676 | 0.597 | 0.682 | 0.444 | 0.420 | 36.5 | 1.97×10^-12^ | 1.50 |

**Table S6.** Performance of PLS models using CHI IAM and log *k*_AGP_

| Model | Components | *R*² | *Q*²_cv_ | *Q*²_ext_ | RMSE_CV_ | RMSEP |
| --- | --- | --- | --- | --- | --- | --- |
| CHI IAM + log *k*_AGP_ | 1 | 0.748 | 0.714 | 0.747 | 0.369 | 0.435 |
| CHI IAM + log *k*_AGP_ | 2 | 0.769 | 0.700 | 0.759 | 0.378 | 0.428 |

**Table S7.** Full performance of all ordinal regression models

| Model | log Lik | AIC | BIC | *R*²* | Accuracy | | | Macro F1 | | | Macro OVR | | |
| --- | --- | --- | --- | --- | --- | --- | --- | --- | --- | --- | --- | --- | --- |
|  |  |  |  |  | Train | CV | Test | Train | CV | Test | Train AUC | CV AUC | Test AUC |
| CHI IAM | -31.5 | 69.1 | 74.9 | 0.440 | 0.692 | 0.692 | 0.769 | 0.689 | 0.687 | 0.778 | 0.876 | 0.860 | 0.923 |
| CHI IAM + *f*_neg_ | -30.5 | 69.0 | 76.8 | 0.458 | 0.731 | 0.731 | 0.769 | 0.734 | 0.730 | 0.778 | 0.882 | 0.848 | 0.906 |
| CHI IAM + log *k*_AGP_ | -30.2 | 68.4 | 76.2 | 0.463 | 0.692 | 0.654 | 0.615 | 0.692 | 0.656 | 0.588 | 0.886 | 0.838 | 0.940 |
| log k_AGP_ | -35.0 | 76.0 | 81.9 | 0.377 | 0.692 | 0.673 | 0.769 | 0.692 | 0.670 | 0.761 | 0.837 | 0.823 | 0.861 |
| log *k*_AGP_ + *f*_pos_ | -30.9 | 69.8 | 77.6 | 0.451 | 0.769 | 0.692 | 0.692 | 0.777 | 0.697 | 0.652 | 0.886 | 0.867 | 0.829 |
| log *k*_HSA_ | -48.7 | 103 | 109 | 0.134 | 0.558 | 0.538 | 0.615 | 0.555 | 0.534 | 0.622 | 0.723 | 0.704 | 0.795 |
| log *k*_HSA_ + *f*_pos_ | -37.4 | 82.9 | 90.7 | 0.334 | 0.654 | 0.654 | 0.538 | 0.653 | 0.646 | 0.509 | 0.823 | 0.794 | 0.839 |
| log *k*_HSA_ + *f*_pos_ + *f*_neg_ | -34.1 | 78.2 | 88.0 | 0.394 | 0.673 | 0.635 | 0.615 | 0.675 | 0.630 | 0.613 | 0.848 | 0.808 | 0.871 |
| log *D*_7.4_ | -46.8 | 99.5 | 105 | 0.169 | 0.519 | 0.558 | 0.692 | 0.522 | 0.555 | 0.689 | 0.741 | 0.721 | 0.915 |
| log *D*_7.4_ + *f*_pos_ | -37.9 | 83.7 | 91.5 | 0.327 | 0.596 | 0.596 | 0.615 | 0.599 | 0.596 | 0.613 | 0.828 | 0.801 | 0.898 |
| log *P* + *f*_pos_ | -36.1 | 80.2 | 88.0 | 0.358 | 0.596 | 0.596 | 0.692 | 0.598 | 0.590 | 0.689 | 0.842 | 0.806 | 0.855 |
| log *P* | -41.5 | 89.0 | 94.9 | 0.262 | 0.654 | 0.654 | 0.615 | 0.644 | 0.644 | 0.588 | 0.796 | 0.764 | 0.847 |

*McFadden’s *R*^2^

**Table S8.** Coefficients of ordinal regression models

| Model | Term | Ordinal logit coefficient (*β*) | Std. Error | *z*-value | *p*-value | 95% CI low | 95% CI high |
| --- | --- | --- | --- | --- | --- | --- | --- |
| CHI IAM | CHI IAM | 0.198 | 0.0427 | 4.64 | 3.50×10^-6^ | 0.114 | 0.282 |
| CHI IAM + *f*_neg_ | CHI IAM | 0.188 | 0.0432 | 4.35 | 1.37×10^-5^ | 0.103 | 0.272 |
|  | *f*_neg_ | -1.63 | 1.15 | -1.42 | 0.156 | -3.88 | 0.624 |
| CHI IAM  + log *k*_AGP_ | CHI IAM | 0.143 | 0.0517 | 2.77 | 0.00567 | 0.0417 | 0.244 |
|  | log *k*_AGP_ | 1.38 | 0.885 | 1.56 | 0.119 | -0.354 | 3.11 |
| log *k*_AGP_ | log *k*_AGP_ | 3.15 | 0.742 | 4.24 | 2.19×10^-5^ | 1.70 | 4.61 |
| log *k*_AGP_  + *f*_pos_ | log *k*_AGP_ | 3.33 | 0.845 | 3.95 | 7.94×10^-5^ | 1.68 | 4.99 |
|  | *f*_pos_ | 2.99 | 1.14 | 2.63 | 0.00866 | 0.757 | 5.22 |
| log *k*_HSA_ | log *k*_HSA_ | 1.43 | 0.408 | 3.51 | 4.51×10^-4^ | 0.631 | 2.23 |
| log *k*_HSA_ + *f*_pos_ | log *k*_HSA_ | 2.12 | 0.506 | 4.19 | 2.74×10^-5^ | 1.13 | 3.11 |
|  | *f*_pos_ | 4.59 | 1.16 | 3.95 | 7.90×10^-5^ | 2.31 | 6.87 |
| log *k*_HSA_ + *f*_pos_ + *f*_neg_ | log *k*_HSA_ | 2.32 | 0.568 | 4.08 | 4.53×10^-5^ | 1.20 | 3.43 |
|  | *f*_pos_ | 3.99 | 1.17 | 3.42 | 6.30×10^-4^ | 1.70 | 6.28 |
|  | *f*_neg_ | -2.85 | 1.23 | -2.32 | 0.0203 | -5.26 | -0.444 |
| log *D*_7.4_ | log *D*_7.4_ | 0.569 | 0.150 | 3.79 | 1.53×10^-4^ | 0.275 | 0.864 |
| log *D*_7.4_ + *f*_pos_ | log *D*_7.4_ | 0.718 | 0.179 | 4.01 | 6.00×10^-5^ | 0.367 | 1.07 |
|  | *f*_pos_ | 3.64 | 1.00 | 3.64 | 2.75×10^-4^ | 1.68 | 5.61 |
| log *P* + *f*_pos_ | log *P* | 0.900 | 0.212 | 4.24 | 2.20×10^-5^ | 0.484 | 1.32 |
|  | *f*_pos_ | 2.96 | 0.983 | 3.01 | 0.00259 | 1.03 | 4.89 |
| log *P* | log *P* | 0.866 | 0.196 | 4.42 | 1.00×10^-5^ | 0.482 | 1.25 |

**Table S9.** Cutpoint estimates of ordinal regression models

| Model | Cutpoint | Estimate | Std. Error | *z*-value | *p*-value | 95% CI low | 95% CI high |
| --- | --- | --- | --- | --- | --- | --- | --- |
| CHI IAM | 0/1 | 5.69 | 1.47 | 3.88 | 1.05×10^-4^ | 2.81 | 8.56 |
|  | 1/2 | 1.36 | 0.232 | 5.87 | 4.36×10^-9^ | 0.908 | 1.82 |
| CHI IAM + *f*_neg_ | 0/1 | 4.79 | 1.50 | 3.19 | 0.00141 | 1.85 | 7.73 |
|  | 1/2 | 1.46 | 0.245 | 5.93 | 2.95×10^-9^ | 0.975 | 1.94 |
| CHI IAM  + log *k*_AGP_ | 0/1 | 3.88 | 1.72 | 2.25 | 0.0244 | 0.500 | 7.25 |
|  | 1/2 | 1.45 | 0.245 | 5.92 | 3.27×10^-9^ | 0.969 | 1.93 |
| log *k*_AGP_ | 0/1 | -0.568 | 0.536 | -1.06 | 0.289 | -1.62 | 0.482 |
|  | 1/2 | 1.21 | 0.226 | 5.37 | 7.98×10^-8^ | 0.770 | 1.66 |
| log *k*_AGP_ + *f*_pos_ | 0/1 | 1.37 | 0.924 | 1.48 | 0.139 | -0.443 | 3.18 |
|  | 1/2 | 1.42 | 0.247 | 5.73 | 1.00×10^-8^ | 0.933 | 1.90 |
| log *k*_HSA_ | 0/1 | -0.517 | 0.389 | -1.33 | 0.184 | -1.28 | 0.246 |
|  | 1/2 | 0.748 | 0.206 | 3.64 | 2.74×10^-4^ | 0.345 | 1.15 |
| log *k*_HSA_ + *f*_pos_ | 0/1 | 2.85 | 0.946 | 3.01 | 0.00262 | 0.993 | 4.70 |
|  | 1/2 | 1.15 | 0.224 | 5.13 | 2.90×10^-7^ | 0.711 | 1.59 |
| log *k*_HSA_  + *f*_pos_ + *f*_neg_ | 0/1 | 1.84 | 0.965 | 1.90 | 0.0571 | -0.055 | 3.73 |
|  | 1/2 | 1.30 | 0.236 | 5.53 | 3.24×10^-8^ | 0.842 | 1.77 |
| log *D*_7.4_ | 0/1 | -0.502 | 0.386 | -1.30 | 0.193 | -1.26 | 0.254 |
|  | 1/2 | 0.791 | 0.204 | 3.87 | 1.08×10^-4^ | 0.390 | 1.19 |
| log *D*_7.4_ + *f*_pos_ | 0/1 | 2.05 | 0.852 | 2.41 | 0.0161 | 0.381 | 3.72 |
|  | 1/2 | 1.07 | 0.214 | 5.02 | 5.11×10^-7^ | 0.655 | 1.49 |
| log *P* + *f*_pos_ | 0/1 | 3.15 | 0.985 | 3.20 | 0.00140 | 1.22 | 5.08 |
|  | 1/2 | 1.16 | 0.218 | 5.31 | 1.08×10^-7^ | 0.731 | 1.58 |
| log *P* | 0/1 | 1.12 | 0.610 | 1.83 | 0.0677 | -0.0810 | 2.31 |
|  | 1/2 | 0.961 | 0.210 | 4.58 | 4.57×10^-6^ | 0.550 | 1.37 |

pEC_50_

**Figure S1.** Distribution of pEC₅₀ values in the training and external test sets, demonstrating the preservation of activity range and representativeness between the two subsets

Contribution of compounds, %

**Figure S2.** Distribution of compounds across charge classes in the training and test sets, showing consistent representation of ionization states following ordinal dataset splitting

**Figure S3.** Training-set confusion matrix for the ordinal regression model based on CHI IAM. Misclassifications occurred mainly between adjacent phospholipidosis classes, with no direct confusion between non-inducers and strong inducers

**Figure S4.** Training-set multiclass receiver operating characteristic curves for the CHI IAM ordinal regression model. Curves were generated using a one-vs-rest approach, in which each PLD class was treated as the positive class, and the remaining two classes were combined as the negative group

**References**

[1] L. LeCureux, C.S. Cheng, J. Herbst, et al. Evaluation and validation of multiple cell lines and primary mouse macrophages to predict phospholipidosis potential. *Toxicology in Vitro* **25** (2011) 1934-1943. <https://dx.doi.org/10.1016/j.tiv.2011.06.017>
[2] S.A. Shahane, R. Huang, D. Gerhold, et al. Detection of phospholipidosis induction: a cell-based assay in high-throughput and high-content format. *SLAS Discovery* **19** (2014) 66-76. <https://dx.doi.org/10.1177/1087057113502851>
[3] F. Atienzar, H. Gerets, S. Dufrane, et al. Determination of phospholipidosis potential based on gene expression analysis in HepG2 cells. *Toxicological Sciences* **96** (2007) 101-114. <https://dx.doi.org/10.1093/toxsci/kfl184>
